# Venetoclax combinations delay the time to deterioration of HRQoL in unfit patients with acute myeloid leukemia

**DOI:** 10.1038/s41408-022-00668-8

**Published:** 2022-04-20

**Authors:** Keith W. Pratz, Panayiotis Panayiotidis, Christian Recher, Xudong Wei, Brian A. Jonas, Pau Montesinos, Vladimir Ivanov, Andre C. Schuh, Courtney D. DiNardo, Jan Novak, Vlatko Pejsa, Don Stevens, Su-Peng Yeh, Inho Kim, Mehmet Turgut, Nicola Fracchiolla, Kazuhito Yamamoto, Yishai Ofran, Andrew H. Wei, Cat N. Bui, Katy Benjamin, Rajesh Kamalakar, Jalaja Potluri, Wellington Mendes, Jacob Devine, Walter Fiedler

**Affiliations:** 1 Abramson Cancer Center, University of Pennsylvania, Philadelphia, PA USA; 2 National and Kapodistrian University of Athens Medical School, Laiko General Hospital, Athens, Greece; 3 Service d’Hématologie, Centre Hospitalier Universitaire de Toulouse, Institut Universitaire du Cancer de Toulouse Oncopole, Université de Toulouse 3 Paul Sabatier, Toulouse, France; 4 The Affiliated Cancer Hospital of Zhengzhou University/Henan Cancer Hospital, Zhengzhou, China; 5 Department of Internal Medicine, Division of Hematology and Oncology, University of California Davis School of Medicine, Sacramento, CA USA; 6 Hospital Universitario y Politécnico La Fe, Valencia, Spain; 7 Almazov National Medical Research Center, Saint Petersburg, Russian Federation; 8 Princess Margaret Cancer Centre and University of Toronto, Toronto, ON Canada; 9 Department of Leukemia, Division of Cancer Medicine, The University of Texas MD Anderson Cancer Center, Houston, TX USA; 10 Department of Internal Medicine and Hematology, University Hospital Kralovske Vinohrady and Third Faculty of Medicine, Charles University, Prague, Czech Republic; 11 Department of Hematology, University Hospital Dubrava, University of Zagreb School of Medicine, Zagreb, Croatia; 12 Norton Cancer Institute, Louisville, KY USA; 13 Department of Internal Medicine, China Medical University Hospital, Taichung, Taiwan; 14 Seoul National University Hospital, Seoul, Republic of Korea; 15 Department of Internal Medicine, Division of Hematology, Ondokuz Mayıs University Faculty of Medicine, Samsun, Turkey; 16 Hematology Unit, Fondazione IRCCS Ca’ Granda-Ospedale Maggiore Policlinico, Milan, Italy; 17 Department of Hematology and Cell Therapy, Aichi Cancer Center, Nagoya, Japan; 18 Department of Hematology, Shaare Zedek Medical Center, Faculty of Medicine, Hebrew University of Jerusalem, Jerusalem, Israel; 19 Australian Center for Blood Diseases, The Alfred Hospital and Monash University, Melbourne, Australia; 20 AbbVie Inc., North Chicago, IL USA; 21 Genentech Inc., South San Francisco, CA USA; 22 Department of Oncology, Hematology and Bone Marrow Transplantation With Section Pneumology, Hubertus Wald University Cancer Center, University Medical Center Hamburg-Eppendorf, Hamburg, Germany

**Keywords:** Quality of life, Cancer

## Abstract

Phase 3 trials Viale-A and Viale-C evaluated health-related quality of life (HRQoL) in patients with AML unfit for intensive chemotherapy who received venetoclax (VEN) + (AZA) (Viale-A) or low-dose cytarabine (LDAC) (Viale-C) or placebo (PBO) + AZA or LDAC. Patient-reported outcomes included: EORTC QLQ-C30 global health status (GHS/QoL) and physical functioning (PF), PROMIS Cancer Fatigue Short Form 7a (Fatigue), and EQ-5D-5L health status visual analog scale (HS-VAS). Time to deterioration (TTD), defined as worsening from baseline in meaningful change thresholds (MCT) of ≥10, 5, or 7 points for GHS/QoL or PF, fatigue, and HS-VAS, respectively, was assessed; differences between groups were analyzed using Kaplan-Meier and unadjusted log-rank analyses. VEN + AZA vs PBO + AZA patients had longer TTD in GHS/QoL (*P* = 0.066) and fatigue (*P* = 0.189), and significantly longer TTD in PF (*P* = 0.028) and HS-VAS (*P* < 0.001). VEN + LDAC vs PBO + LDAC patients had significantly longer TTD in GHS/QoL (*P* = 0.011), *P*F (*P* = 0.020), and fatigue (*P* = 0.004), and a trend in HS-VAS (*P* = 0.057). Approximately 43%, 35%, 32%, and 18% of patients treated with VEN + AZA, AZA + PBO, VEN + LDAC, or LDAC + PBO, respectively, saw improvements >MCT in GHS/QoL. Overall, VEN may positively impact HRQoL in patients with AML ineligible for intensive chemotherapy, leading to longer preservation of functioning and overall health status.

## Introduction

Acute myeloid leukemia (AML) is an aggressive myeloid neoplasm that has a poor prognosis, with a reported 5-year overall survival rate estimated at 29% in recent historical series, and less than 10% in series of older adults [1]. The standard-of-care first-line treatment for patients with AML is intensive chemotherapy including cytarabine and an anthracycline [2]. Recent advances in intensive therapy include the use of midostaurin and gemtuzumab ozogamicin in association with induction chemotherapy in specific subsets of patients [3, 4]. Many patients in older age groups may not be eligible for intensive chemotherapy, however, due to factors such as age, comorbidities, or unfavorable genomic features [2, 5, 6]. Such patients often have poorer prognoses [7], and are commonly treated with less intensive regimens, which may include hypomethylating agents (ie, azacitidine or decitabine) and low-dose cytarabine (LDAC) [8–12]. The Food and Drug Administration (FDA) recently approved venetoclax in combination with azacitidine, decitabine, or LDAC for the treatment of newly diagnosed patients with AML who are ineligible for intensive chemotherapy, with these regimens representing new standards of care, due to improvements in response rates and overall survival [13, 14].

An important goal of treatment in all patients is to prolong health-related quality of life (HRQoL). Venetoclax in combination with azacitidine or LDAC for patients who are ineligible for intensive chemotherapy has been shown to be effective in increasing both complete remission rate and overall survival [15–20], and yet the impact of venetoclax as a combination therapy on the HRQoL of patients is not well known. Patients with AML have reduced QoL, including reductions in physical functioning (PF), increased fatigue, and decreased ability to maintain social and daily activities [21–23], with fatigue reported as the most burdensome symptom irrespective of treatment status [24]. At baseline, patients with AML who are ineligible for intensive chemotherapy had poor HRQoL scores, particularly in EORTC global health status (GHS) (median score of 50) and fatigue (median score of 53–66) on a 0–100 scale [25–27]. Furthermore, baseline impairments in role and physical domains, health status, and fatigue are lower than those observed in the general population, and QoL tends to deteriorate quickly at the time of diagnosis and treatment initiation [22, 24]. The impact of treatment on patient-reported outcomes (PROs) in patients with AML or other leukemias is increasingly appreciated as an important clinical outcome when treatment benefits are evaluated [24, 28], and thus can aid patients and providers in clinical decision making [29, 30].

Treatment of AML, even in unfit patients receiving less aggressive therapies, is associated with events that may negatively impact HRQoL including infection-related complications, high transfusion needs, and hospitalization [24]. The importance of capturing potential treatment benefits and outcomes from the patient perspective is further highlighted by an observed discordance between how patients and physicians perceive the patients’ HRQoL [31]. This study aimed to comprehensively characterize HRQoL outcomes of newly diagnosed patients with AML, and particularly potential delay in deterioration of HRQoL, for patients treated with venetoclax or placebo in the Viale-A and Viale-C clinical trials.

## Materials and methods

### Study design and patients

Full details on study design and eligibility criteria for the Viale-A (NCT02993523) and Viale-C (NCT03069352) Phase 3 clinical trials have been previously published [15, 18]. This secondary analysis utilized PRO data collected from these studies. Briefly, Viale-A and Viale-C are randomized, double-blind, placebo controlled, multicenter trials in patients ≥18 years of age with a confirmed diagnosis of untreated AML who were ineligible for intensive chemotherapy due to age or pre-defined comorbidities.

As previously described, patients in Viale-A were randomized 2:1 to receive venetoclax 400 mg orally once daily (QD) or placebo for 28 days, in combination with azacitidine 75 mg/m^2^ subcutaneous (SC) or intravenous (IV) for 7 days [15]. Patients in Viale-C were randomized 2:1 to receive venetoclax 600 mg orally QD or placebo for 28 days, in combination with LDAC 20 mg/m^2^ SC QD for 10 days [18]. Patients continued their assigned treatments in 28-day cycles, until documented disease progression per investigator assessment, unacceptable toxicity, withdrawal of consent, or other protocol criteria for discontinuation (whichever occurred first).

### Ethics

For both trials, local ethics committee approvals were obtained, and patients included in the trials provided written informed consent. The studies were conducted in accordance with the International Conference on Harmonisation, Good Clinical Practice guidelines, and the Declaration of Helsinki.

### Patient-reported outcomes

Patients with AML may experience deteriorations in HRQoL over time for multiple causes, including treatment-related complications or disease progression; this analysis assessed whether the addition of venetoclax to either azacitidine or LDAC would prolong the time to such deterioration. Several validated PRO instruments were utilized in the Viale-A and Viale-C trials to capture the patients’ experience regarding treatment benefits across various key HRQoL domains important to this patient population; instruments used included the European Organisation for Research and Treatment of Cancer Quality of Life Questionnaire Core [EORTC QLQ-C30] global health status (GHS/QoL) and PF scales, the Patient Reported Outcomes Measurement Information System, the Cancer Fatigue Short Form SF 7a (PROMIS Fatigue SF 7A), and the EuroQoL 5 Dimension 5 level (EQ-5D-5L) visual analog scale (health status VAS) [32–36].

The EORTC QLQ-C30 comprises 30 items addressing 15 HRQoL domains, including five multi-item functional scales (physical, emotional, cognitive, social, and role functioning), three multi-item symptom scales (fatigue, nausea and vomiting, and pain), six single-item symptom scales (dyspnea, insomnia, appetite loss, constipation, diarrhea, and financial difficulties) and a GHS/QoL scale. All scales are scored on a 0–100 metric. For GHS/QoL and functional scales, a high score represents higher/better level of functioning and for symptom scales a higher score indicates worse symptom severity. The recall period for EORTC QLQ-C30 is the past week. The PROMIS Fatigue SF 7a or fatigue has been developed for use in oncology and other chronic disease populations [33] and consists of a seven-item questionnaire assessing the impact and experience of fatigue over the past 7 days with higher scores representing worse fatigue. The EQ-5D-5L VAS or health status VAS, which measures current overall health status, is scored on a scale of 0–100 where 100 represents “the best health you can imagine,” and 0 indicates “the worst health you can imagine.”

All PRO questionnaires were collected on Cycle 1 Day 1, and then on Day 1 of every other cycle throughout both trials, including the final visit. The primary outcome of this analysis was to compare the delay in time to deterioration for each PRO measure, for patients randomized to venetoclax in combination with azacitidine or LDAC to those treated with azacitidine or LDAC. Deterioration in HRQoL was defined as the worsening from baseline in PRO-specific meaningful change thresholds (MCTs). Time to deterioration was assessed overall and for patients in key subgroups. Subgroups analyzed included patients with complete remission + complete remission and incomplete blood cell recovery (CR + CRi), ages <75 or ≥75 years, defined by postbaseline transfusion independence (TI) status (RBC or platelets), or with baseline ECOG score >2. An additional analysis was conducted to compare the proportions of patients in each treatment group who reported improvements from baseline in at least one MCT for each PRO measure. MCTs were defined based upon published thresholds of change of ≥10, and 7 points for GHS/QoL and PF [37], and health status VAS [38], respectively. In this study, MCT was defined as a change of ≥5 points for fatigue.

### Statistical analysis

Time to deterioration was summarized as the median and 95% confidence interval (CI) for each treatment group. Kaplan-Meier curves were calculated, as well as the unadjusted log-rank *P-value* for the difference between treatment arms. Cox proportional hazards regression models adjusted for key covariates (age, baseline Eastern Cooperative Oncology Group and PRO scores, AML type, and cytogenetic risk category); hazard ratios (HR) and their corresponding 95% CIs comparing the treatment groups are reported. The proportion of patients who reported improvements ≥ MCTs for a specified PRO at any point post-baseline was reported as numbers and percentages. Improvement in HRQoL was defined as an improvement in score from baseline of at least one PRO-specific MCT at any time. Improvements in HRQoL were assessed overall and for patients who achieved CR + CRi.

Time to deterioration was calculated as the number of days from baseline to the date of the first observed deterioration event, defined as a decline from baseline score of at least one MCT for each PRO at a post-baseline assessment among patients with available data on the PRO measure at baseline. Patients without a baseline PRO measure were excluded from analysis. Deterioration events were based on the change from the baseline score only; therefore, any deterioration of PRO scores between follow-up visits (i.e, improvement and then worsening) was not captured in this analysis even if this decline was ≥1 MCT between visits. If a specified deterioration event did not occur, patients were censored at the last observed post-baseline PRO assessment. If the post-baseline PRO measure was not available, then the patient was censored a day after the baseline PRO assessment date (censored at baseline + 1 day). Analyses were based upon the longitudinal analysis population, comprising all patients in the full analysis dataset (intention-to-treat population) who survived up to a given assessment and had available data on at least one PRO measure at baseline and for that assessment. The number of patients available for analysis is reflected in the Kaplan Meier plots as the N number of patients at risk at month 0.

### Sensitivity analysis

A sensitivity analysis was utilized to assess time to deterioration from baseline PRO assessment to a composite endpoint of progressive disease, death, or symptom worsening, defined as a change in score of at least one MCT in a relevant PRO measure. Deterioration was determined by the first occurrence of any one of these events and if none of the specified events occurred, or if patients did not have any post-baseline PRO assessments, their data were censored at the last observed disease assessment date (same censoring date for event-free survival). Patients without a baseline PRO assessment were excluded from the sensitivity analysis.

## Results

### Baseline demographics and clinical characteristics

Viale-A included 431 pts (venetoclax + azacitidine: 286, placebo + azacitidine: 145) and Viale-C included 211 pts (venetoclax + LDAC: 143, placebo + LDAC: 68). In both studies and in all treatment groups, the median age was 76 years and nearly 60% patients were male. In Viale-A, secondary AML has reported in 25% and 24% and poor cytogenetic risk in 36% and 39% of patients in the venetoclax + azacitidine and placebo + azacitidine groups, respectively. In Viale-C, secondary AML was reported in 41% and 34% and poor cytogenetic risk in 29% and 33% of patients in the venetoclax + LDAC and placebo + LDAC groups, respectively (Supplemental Table 1).

### Viale-A time to deterioration in HRQoL

**Fig. 1:**
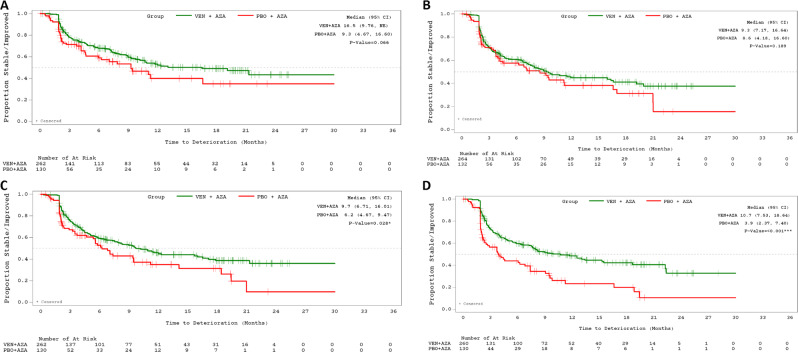
Time to deterioration of PROs for Azacitidine based patients. Time to deterioration in EORTC GHS/QoL (**A**), PROMIS Fatigue (**B**), EORTC PF (**C**), and Health Status (EQ-5D-5L) VAS (**D**), VEN + AZA versus PBO + AZA. Time to deterioration thresholds for EORTC-QLQ-C30, EQ-5D-5L VAS, and PROMIS Fatigue are ≥10, 7, or 5 points, respectively. Time to deterioration analyses were conducted for all patients in the full dataset with available data on ≥1 PRO measures from baseline to the given assessment (*N* = VEN + AZA 262, 264, 262, and 260, and PBO + AZA 130, 132, 130, and 130 for EORTC GHS/QoL, fatigue, PF, and health status VAS, respectively). AZA azacitidine, CI confidence interval, EORTC QLQ-C30 European Organization for Research and Treatment of Cancer quality of life questionnaire, EQ-5D-5L EuroQoL 5-Dimension 5-Level, GHS global health status, NE not estimable, PBO placebo, PF physical functioning, PROMIS Patient-Reported Outcomes Measurement Information System, QoL quality of life, VAS visual analog scale, VEN venetoclax.

Compared with placebo + azacitidine patients, venetoclax + azacitidine patients showed a trend towards longer time to deterioration in GHS/QoL (median in months: 16.5 vs. 9.3, *P* = 0.066) and fatigue (9.3 vs. 8.6, *P* = 0.189) (Fig. 1; Table 1). Venetoclax + azacitidine patients had a significantly longer time to deterioration in PF (9.7 vs. 6.2, *P* = 0.028) and health status VAS (10.7 vs. 3.9, *P* < 0.001) than did patients treated with placebo + azacitidine (Fig. 1; Table 1).

**Table 1:** Cox proportional hazards models: median time (months) to deterioration in overall health/QoL, physical function, and fatigue, Viale‑A and Viale-C.

PRO measure	Viale-A			Viale-C		
	VEN + AZA (*n* = 286)	PBO + AZA (*n* = 145)	HR (95% CI)	VEN + LDAC (*n* = 143)	PBO + LDAC (*n* = 68)	HR (95% CI)
EORTC QLQ-C30 GHS/QoL	16.5 (9.8, NE)	9.3 (4.7, 16.6)	0.81 (0.55, 1.2)	11.3 (4.2, NE)	2.6 (2.0, 9.3)	0.37 (0.21, 0.64)**
EORTC QLC-C30 PF	9.7 (6.7, 16.0)	6.2 (4.7, 9.5)	0.63 (0.45, 0.88)*	5.8 (3.1, NE)	2.9 (2.0, 8.1)	0.43 (0.26, 0.72)*
PROMIS Fatigue	9.3 (7.2, 16.6)	8.6 (4.2, 16.6)	0.72 (0.51, 1.0)	8.1 (5.8, NE)	2.6 (2.1, 9.5)	0.37 (0.21, 0.65)**
EQ-5D-5L Health status VAS	10.7 (7.5, 18.6)	3.9 (2.4, 7.4)	0.55 (0.39, 0.77)**	4.9 (2.8, NE)	2.5 (2.0, 9.5)	0.49 (0.29, 0.81)*

*P < 0.01. **P < 0.001. *AZA* azacitidine, CI confidence interval, EORTC QLQ-C30 European Organization for Research and Treatment of Cancer quality of life questionnaire, EQ-5D-5L EuroQoL 5-Dimension 5-Level, GHS global health status, HR hazard ratio, LDAC low-dose cytarabine, NE not estimable, PBO placebo, PF physical functioning, PRO patient-reported outcome, PROMIS Patient-Reported Outcomes Measurement Information System, QoL quality of life, VAS visual analog scale, VEN venetoclax.

### Viale-C time to deterioration in HRQoL

**Fig. 2:**
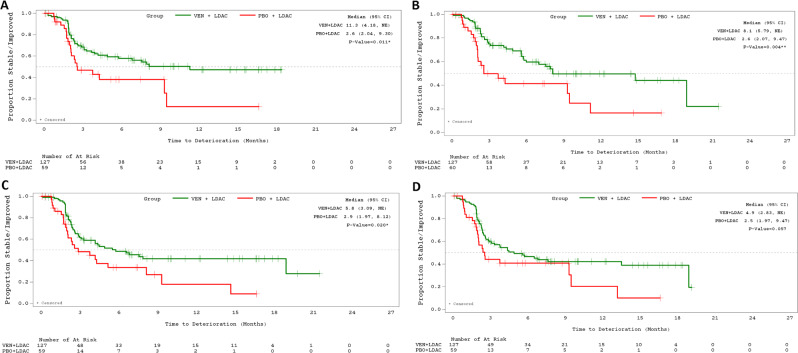
Time to deterioration of PROs for LDAC based patients. Time to deterioration in EORTC GHS/QoL (**A**), PROMIS Fatigue (**B**), EORTC PF (**C**), and Health Status (EQ-5D-5L) VAS (**D**), VEN + LDAC versus PBO + LDAC. Time to deterioration thresholds for EORTC-QLQ-C30, EQ-5D-5L VAS, and PROMIS Fatigue are ≥10, 7, or 5 points, respectively. Time to deterioration analyses were conducted for all patients in the full dataset with available data on ≥1 PRO measures from baseline to the given assessment (*N* = VEN + LDAC: 127, 127, 127, and 127; and PBO + LDAC: 59, 60, 59, and 59 for EORTC GHS/QoL, fatigue, PF, and health status VAS, respectively). CI confidence interval, EORTC QLQ-C30 European Organization for Research and Treatment of Cancer quality of life questionnaire, EQ-5D-5L EuroQoL 5-Dimension 5-Level, GHS global health status, LDAC low-dose cytarabine, NE not estimable, PBO placebo, PF physical functioning, PROMIS Patient-Reported Outcomes Measurement Information System, QoL quality of life, VAS visual analog scale, VEN venetoclax.

Compared with those in the placebo + LDAC group, venetoclax + LDAC patients experienced a significantly longer time to deterioration in GHS/QoL (11.3 vs. 2.6, *P* = 0.011), PF (5.8 vs. 2.9, *P* = 0.020), and fatigue (8.1 vs. 2.6, *P* = 0.004), and a trend towards longer time to deterioration in health status VAS (4.9 vs. 2.5, *P* = 0.057) (Fig. 2; Table 1).

### Cox regression model

Cox proportional hazards models demonstrated consistently longer time to deterioration in all PRO-assessed measures for patients treated with venetoclax as compared with placebo in both trials, with significant differences for PF and health status VAS in Viale-A, and for all measures in Viale-C (*P* ≤ 0.01) (Table 1).

### Subgroup analyses

There was a trend towards longer time to deterioration in the venetoclax + azacitidine group versus the placebo + azacitidine group for all subgroups for GHS/QoL and in health status VAS for the CR + CRi subgroup (Supplemental Fig. 1–6). Similar results were observed in the Viale-C trial for all subgroups except for CR + Cri, where the sample size of patients attaining CR + CRi was too small in the control patient population to allow robust between treatment group statistical analysis. There was a trend towards longer time to deterioration in GHS/QoL observed in venetoclax treated patients in the subgroup of patients <75 years of age, which was significant in patients treated in Viale-A (Table 2); notably, in patients >75 years of age, there was a similar time to deterioration between the treatment groups.

**Table 2:** Median (95% CI) time (months) to deterioration in general QoL PROs key subgroup analyses.

PRO measure, median month (95% CI)	Viale-A				Viale-C			
	VEN + AZA (*n* = 286)		PBO + AZA (*n* = 145)		VEN + LDAC (*n* = 143)		PBO + LDAC (*n* = 68)	
CR + CRi								
EORTC QLQ-C30 GHS/QoL	21.3 (11.7, NE)		16.6 (5.6, NE)		NE (8.12, NE)		Small N in control arm†	
EQ-5D-5L Health status VAS	13.4 (8.0, 22.4)		7.4 (3.7, 19.4)		18.9 (6.81, NE)		Small N in control arm†	
Age < 75 years								
EORTC QLQ-C30 GHS/QoL	19.1 (10.2, NE)		4.7 (2.2, 16.6)*		NE (5.8, NE)		4.3 (1.7, NE)	
Age > = 75 years								
EORTC QLQ-C30 GHS/QoL	12.0 (7.8, NE)		11.2 (7.1, NE)		6.8 (3.1, NE)		2.4 (1.8, 9.3)	
Postbaseline TI RBC								
EORTC QLQ-C30 GHS/QoL	21.3 (10.9, NE)		16.6 (6.0, NE)		NE (7.9, NE)		9.5 (1.8, NE)	
Postbaseline TI Platelet								
EORTC QLQ-C30 GHS/QoL	19.1 (10.7, NE)		11.0 (4.7, NE)		NE (5.8, NE)		9.3 (2.5, NE)	
Baseline ECOG score > 2								
EORTC QLQ-C30 GHS/QoL	19.1 (9.2, NE)		7.9 (2.7, NE)		8.1 (3.7, NE)		4.3 (1.7, 9.5)	

*P < 0.01.

†N < 10 patients.

*AZA* azacitidine, *CI* confidence interval, *CR* complete remission, *CRi* complete remission with incomplete blood count recovery, *EORTC QLQ-C30* European Organization for Research and Treatment of Cancer quality of life questionnaire, *EQ-5D-5L* EuroQoL 5-Dimension 5-Level, *GHS* global health status, *LDAC* low-dose cytarabine, *NE* not estimable, *PBO* placebo, *PRO* patient-reported outcome, *QoL* quality of life, *RBC* red blood cells, *TI* transfusion independence, *VAS* visual analog scale, *VEN*, venetoclax.

### Percent improvements in HRQoL

Across all PROs, numerically greater proportions of patients treated with venetoclax + azacitidine or LDAC versus placebo + azacitidine or LDAC reported improvements at any time post-baseline (Table 3). Specifically, 43% versus 35% of patients treated with venetoclax + azacitidine versus placebo + azacitidine and 32% versus 18% of patients treated with venetoclax + LDAC versus placebo + LDAC reported improvements in GHS/QoL. Compared with placebo + azacitidine, greater proportions of patients treated with venetoclax + azacitidine reported improvements in PF (29% vs. 26%), fatigue (39% vs. 32%), and health status VAS (43% vs. 27%). Compared with placebo + LDAC, greater proportions of patients treated with venetoclax + LDAC reported improvements in PF (32% vs. 16%), fatigue (38% vs. 18%), and health status VAS (33% vs. 22%). Of the total patients who reported improvements, ≥65% reported improvements by cycle 4 for all PROs regardless of treatment group.

**Table 3:** Proportion of patients with improvements at any time post-baseline in PROs overall and by patients achieving CR + Cri.

PRO measure, *N* (%)	Viale-A		Viale-C	
	VEN + AZA (*n* = 286)	PBO + AZA (*n* = 145)	VEN + LDAC (*n* = 143)	PBO + LDAC (*n* = 68)
Overall				
EORTC QLQ-C30 GHS/QoL	123 (43.0)	50 (34.5)	46 (32.2)	12 (17.6)
EORTC QLC-C30 PF	83 (29.0)	37 (25.5)	46 (32.2)	11 (16.2)
PROMIS Fatigue	111 (38.8)	46 (31.7)	54 (37.8)	12 (17.6)
EQ-5D-5L Health status VAS	122 (42.7)	36 (26.9)	47 (32.9)	15 (22.1)
CR + CRi subgroup*				
EORTC QLQ-C30 GHS/QoL				
Yes	108 (37.8)	23 (15.9)	33 (23.1)	3 (4.4)
No	15 (5.2)	27 (18.6)	13 (9.1)	9 (13.2)
EORTC QLC-C30 PF				
Yes	77 (26.9)	22 (15.2)	32 (22.4)	5 (7.4)
No	6 (2.1)	15 (10.3)	14 (9.8)	6 (8.8)
PROMIS Fatigue				
Yes	94 (32.9)	23 (15.9)	40 (28.0)	6 (8.8)
No	17 (5.9)	23 (15.9)	14 (9.8)	6 (8.8)
EQ-5D-5L Health status VAS				
Yes	108 (37.8)	18 (12.4)	35 (24.5)	7 (10.3)
No	14 (4.9)	21 (14.5)	12 (8.4)	8 (11.8)

Improvement thresholds for EORTC-QLQ-C30, EQ-5D-5L VAS, and PROMIS Fatigue are ≥10, 7, or 5 points, respectively.

*Percentages reported are based on the overall study population sample size as the denominator not the subgroup of CR/CRi patients only.

*AZA* azacitidine, *CI* confidence interval, *CR* complete remission, *CRi* complete remission with incomplete blood count recovery, *EORTC QLQ-C30* European Organization for Research and Treatment of Cancer quality of life questionnaire, *EQ-5D-5L* EuroQoL 5-Dimension 5-Level, *GHS* global health status, *LDAC* low-dose cytarabine, *PROMIS* Patient-Reported Outcomes Measurement Information System, *QoL* quality of life, *VAS* visual analog scale, *VEN* venetoclax.

Similar findings were observed among patients who achieved CR + CRi (Table 3). Additionally, greater proportions of patients achieving versus not achieving CR + CRi reported improvements across all PROs among patients who received venetoclax in combination with azacitidine or LDAC.

### Sensitivity analyses

The sensitivity analyses demonstrated that in both Viale-A and Viale-C, the median time to deterioration was significantly longer for patients in the venetoclax + azacitidine or LDAC treatment groups for all instruments and subscales trials (Supplemental Figures 7, 8) than it was for patients receiving placebo + azacitidine or LDAC. However, the inclusion of progression and death in the definition of deterioration contributed to shorter median times to deterioration for all treatment groups in both studies.

## Discussion

Understanding HRQoL from the patient perspective is important in evaluating treatment efficacy, particularly for oncology patients for whom survival may be limited and quality of life during the remaining lifespan is of primary importance to patients, and thus is crucial in treatment decision making, in addition to more standard response-based endpoints [39]. Our study is one of the few to assess longitudinal changes in HRQoL in patients with AML ineligible for intensive chemotherapy within a clinical trial population [10, 26, 40–42]. In our study, significantly longer time to deterioration for patients receiving combination venetoclax compared with placebo + azacitidine or LDAC was observed for all PRO measures, including general QoL and health status, PF, and fatigue in Viale-C, and for PF and health status VAS scores in Viale-A. Interestingly, in Viale-A, the time to deterioration in fatigue was similar in both treatment groups, compared with Viale-C, where patients in the venetoclax + LDAC group experienced a significantly longer time to deterioration compared with the placebo + LDAC group. One interpretation could be that azacitidine may cause treatment-related fatigue that is not ameliorated in these patients. Furthermore, numerically greater proportions of patients receiving combination venetoclax compared with placebo + azacitidine or LDAC reported improvements in GHS/QoL and health status VAS in Viale-A, and GHS/QoL and fatigue in Viale-C. Although the venetoclax combination groups represent a more intensive combination therapy, in this study there was no increased worsening of HRQoL among patients treated with venetoclax + azacitidine or LDAC compared to azacitidine or LDAC alone.

While the effects of non-intensive chemotherapy-based AML therapies on clinical endpoints such as overall survival and relapse-free survival are known [12], their effects on HRQoL remain largely undefined [26]. Key aspects of patient HRQoL impacted by AML include fatigue, PF, anxiety/mental health, and ability to maintain social and role functioning [21, 23]. Patient characteristics may influence HRQoL and treatment-related outcomes, especially among older patients. However, data regarding HRQoL in such patients are limited, and more studies are needed that not only incorporate PROs in older patients, but also track changes in their symptoms and functioning over time [43]. In the current study, patients treated with venetoclax experienced significantly longer time to deterioration compared with those in the placebo group in several HRQoL domains, including overall health status/QoL, PF, and fatigue. These results were largely confirmed in the sensitivity analyses that expanded the definition to include progression, death, and relapse following remission. In this composite analysis, significantly longer time to deterioration was observed in both venetoclax combination groups versus the placebo + azacitidine or LDAC groups across all HRQoL measures, although the inclusion of progression and death in the definition of deterioration contributed to shorter median time to deterioration for all treatment groups in both studies. Overall, the addition of venetoclax trended towards a longer preservation of HRQoL across various measures. In addition to preservation of HRQoL, there were greater proportions of patients reporting improvements in PRO measures of at least one MCT in both venetoclax combination groups versus the placebo + azacitidine or LDAC groups across all HRQoL measures. In a previous analysis, by cycle 2 the percent change from baseline in EORTC QLQ 30 GHS/QoL went up by approximately 30% among patients treated with decitabine versus best supportive care; this change was carried through cycle 4 [44]. Furthermore, in a previously published study, the mean change from baseline in EORTC QLQ 30 GHS/QoL and fatigue scores demonstrated improvements by cycle 3 that were sustained by cycle 9 among patients who received venetoclax + LDAC [18]. Similarly in our study, most patients who reported any improvements in measures of HRQoL from baseline reported them by cycle 4. That HRQoL is not worsened by the addition of venetoclax to a less aggressive strategy such as azacitidine or LDAC alone, may be an added value of combination therapy from the patients’ perspective in treatment decision making, which is further supported by the greater proportions of patients receiving venetoclax combinations versus azacitidine or LDAC alone reporting improvements in HRQoL.

While subgroup analyses were often limited by small sample sizes, which impact the statistical robustness of the results, especially in the control groups and also for later study visits, a subgroup analysis demonstrated consistent prolongation of time to deterioration in the venetoclax combination groups versus placebo groups across various subsets of patients. In the previous clinical efficacy reports, more patients achieved CR/CRi with venetoclax combination treatment compared with the placebo treatment groups [15, 18]. Among patients who were clinical responders (CR + CRi) in the current study, a trend towards longer time to deterioration in GHS/QoL and health status VAS was observed in the venetoclax + azacitidine versus placebo + azacitidine groups in Viale-A. The median time to deterioration was 16.5 months overall and 21.3 months in the CR + CRi subgroup in the venetoclax + azacitidine treatment group. These findings support the longer duration of CR + CRi clinical response observed in the venetoclax + azacitidine (17.5 months) versus placebo + azacitidine (13.4 months) groups in Viale-A [15]. Furthermore, the proportions of patients who reported improvements in HRQoL at any time was numerically higher across all PROs among those who achieved remission versus those who did not in the venetoclax combination arms. These analyses demonstrated that patients achieving remission/response had a similar QoL, suggesting that their HRQoL overall was not compromised by the addition of venetoclax. Previous studies have shown that achievement and durability of remission are factors that may have a role in the extent to which improvements are observed among patients with AML [45, 46]. In Viale-C, distinct separation of KM curves demonstrate a trend of longer time to deterioration in GHS/QoL with the addition of venetoclax versus LDAC alone among patients with post-baseline TI (RBC and platelet); similar results were observed in Viale-A among patients with post-baseline platelet TI. This demonstrates the potential durability of the TI response, which is an indirect measure of QoL as patients may see the reduction in need for transfusions and reduced anemia symptoms as an improvement in their HRQoL. Among patients with baseline ECOG scores >2, there was a trend of longer time to deterioration in general QoL in both venetoclax combination arms versus placebo + azacitidine or LDAC. In younger patients (<75 years) treated with combination venetoclax versus azacitidine, there was a significantly longer time to deterioration in general QoL. In contrast, the time to deterioration was similar between treatment groups in older patients (≥75 years) with a slight trend towards prolongation of HRQoL with venetoclax combination therapy; this finding is important as elderly patients tend to have worse clinical outcomes and HRQoL [7, 43]. A longitudinal study among individuals with AML > 60 years found that QoL improved but remained on similar trajectories with no significant difference in QoL measures observed between those who received intensive or non-intensive treatments, suggesting that similar improvements and/or stability of QoL may be expected regardless of treatment type among older patients [47]. Future studies analyzing the HRQoL during treatment among individuals >75 may be useful to identify areas where combination therapy may impact their HRQoL as part of the treatment plan.

Although the PROs used in this study are validated and commonly used in oncology, there are some limitations to their use [34, 37, 38]. For example, EORTC QLQ-C30 GHS/QoL and EQ-5D-5L health status VAS are generic health measurements not specific to AML. The EORTC QLQ-C30 has been used extensively across multiple types of cancer and has supported labeling claims in the US and Europe [32, 48–50]. Despite this limitation, the PRO measures utilized in this study do contain key aspects that overlap with key domains of HRQoL that were identified as important to patients with AML including fatigue, PF, anxiety, and reductions in the ability to maintain social functioning [21, 23, 51]. However, the lack of distinct separation between the treatment groups observed in this study may suggest that AML-specific measures may be better reflective of the HRQoL of these patients. AML-specific QoL measures are in development and their use in future clinical trials may identify the most appropriate outcomes to measure, leading to a better understanding of the impact of the disease and treatment on patients with AML [51, 52].

One other limitation to the reporting of these data in leukemia is that the primary analysis did not assess improvement of HRQoL from baseline. While the time to deterioration curves did not directly capture improvements, patients who maintained their HRQoL or improved are reflected here indirectly by a longer time to deterioration.

One of the challenges of the PROMIS Fatigue PRO is that patients in all treatment groups in this study received a drug with fatigue as a known side effect. In the Viale-A and Viale-C primary studies, 17–21% and <20% of patients experienced fatigue as an adverse event, respectively [15, 18]. Fatigue is considered a highly burdensome symptom among patients with AML [24], therefore, determining the relevance of PROMIS Fatigue data as compared with adverse event reporting of fatigue is important to address the challenge of separating treatment-related versus disease-related fatigue. PROMIS Fatigue data may be more relevant as the data are patient-reported, providing patients with the opportunity to report their perceived burden of fatigue on their daily activities.

Another limitation is the small sample size beyond the early treatment cycles; however, the early separation of the time to deterioration curves with the initial larger sample size suggests that these results are not due to chance variability and are statistically valid. The small sample sizes in the subgroup analyses limited the interpretation of those results.

In summary, compared with standard chemotherapy alone, venetoclax appears to have a positive impact on the HRQoL, or at least did not significantly worsen it, in patients with AML who are ineligible for intensive chemotherapy, leading to a longer preservation of functioning and overall health status. Between 29% and 43% of patients treated with venetoclax combination therapy saw improvements in their HRQoL, most of these patients reported better scores in their overall health status, physical function, and fatigue by cycle 4. Understanding treatment efficacy from the patient perspective may influence treatment decisions and allow for identification of specific aspects of HRQoL impacted by AML for which strategies may be developed to provide relief. Future studies may utilize AML-specific measurements to further quantify the impact of treatment with venetoclax on PROs.

Supplementary information is available at *Blood Cancer Journal*’s website.

## Supplementary information

Venetoclax combinations delay the time to deterioration of HRQoL in unfit patients with acute myeloid leukemia

## Data Availability

AbbVie is committed to responsible data sharing regarding the clinical trials we sponsor. This clinical trial data can be requested by any qualified researchers who engage in rigorous, independent scientific research, and will be provided following review and approval of a research proposal and Statistical Analysis Plan (SAP)and execution of a Data Sharing Agreement (DSA). Data requests can be submitted at any time and the data will be accessible for12 months, with possible extensions considered. For more information on the process, or to submit a request, visit the following link: https://www.abbvie.com/our-science/clinical-trials/clinical-trials-data-and-information-sharing/data-and-information-sharing-with-qualified-researchers.html.
