# Supplementary material for: Venetoclax combinations delay the time to deterioration of HRQoL in unfit patients with acute myeloid leukemia
Source: Blood Cancer J. 2022 Apr 20;12(4):71. doi: 10.1038/s41408-022-00668-8 (PMC9021259; doi:10.1038/s41408-022-00668-8)
Supplement: Supplementary file 1 — Venetoclax combinations delay the time to deterioration of HRQoL in unfit patients with acute myeloid leukemia [file 41408_2022_668_MOESM1_ESM.docx]

**Venetoclax combinations delay the time to deterioration of HRQoL in unfit patients with acute myeloid leukemia**

Keith W. Pratz, MD^1^, Panayiotis Panayiotidis, MD^2^, Christian Recher, MD, PhD^3^, Xudong Wei, MD, PhD^4^, Brian A. Jonas, MD, PhD^5^, Pau Montesinos, MD, PhD^6^, Vladimir Ivanov, MD, PhD^7^, Andre C. Schuh, MD, FRCPC^8^, Courtney D. DiNardo, MD, MSc^9^, Jan Novak, PhD^10^, Vlatko Pejsa, MD, PhD^11^, Don Stevens, MD^12^, Su-Peng Yeh, MD^13^, Inho Kim, MD, PhD^14^, Mehmet Turgut, MD^15^, Nicola Fracchiolla, MD^16^, Kazuhito Yamamoto, MD, PhD^17^, Yishai Ofran, MD^18^, Andrew H. Wei, MBBS, PhD^19^, Cat N. Bui, PharmD, PhD^20^, Katy Benjamin, MS, PhD^20^, Rajesh Kamalakar, MS^20^, Jalaja Potluri, MD, FACP^20^, Wellington Mendes, MD, PhD^20^, Jacob Devine, MA^21^, Walter Fiedler, MD^22^

^1^Abramson Cancer Center, University of Pennsylvania, Philadelphia, PA, USA; ^2^National and Kapodistrian University of Athens Medical School, Laiko General Hospital, Athens, Greece; ^3^Service d'Hématologie, Centre Hospitalier Universitaire de Toulouse, Institut Universitaire du Cancer de Toulouse Oncopole, Université de Toulouse 3 Paul Sabatier, France; ^4^The Affiliated Cancer Hospital of Zhengzhou University/Henan Cancer Hospital, Zhengzhou, China; ^5^Department of Internal Medicine, Division of Hematology and Oncology, University of California Davis School of Medicine, Sacramento, CA, USA; ^6^Hospital Universitario y Politécnico La Fe, Valencia, Spain; ^7^Almazov National Medical Research Center, Saint Petersburg, Russian Federation; ^8^Princess Margaret Cancer Centre and University of Toronto, Toronto, ON, Canada; ^9^Department of Leukemia, Division of Cancer Medicine, The University of Texas MD Anderson Cancer Center, Houston, TX, USA; ^10^Department of Internal Medicine and Hematology, University Hospital Kralovske Vinohrady and Third Faculty of Medicine, Charles University, Prague, Czech Republic; ^11^Department of Hematology, University Hospital Dubrava, University of Zagreb School of Medicine, Zagreb, Croatia; ^12^Norton Cancer Institute, Louisville, KY, USA; ^13^Department of Internal Medicine, China Medical University Hospital, Taichung, Taiwan; ^14^Seoul National University Hospital, Seoul, Republic of Korea; ^15^Department of Internal Medicine, Division of Hematology, Ondokuz Mayıs University Faculty of Medicine, Samsun, Turkey; ^16^Hematology Unit, Fondazione IRCCS Ca’ Granda-Ospedale Maggiore Policlinico, Milan, Italy; ^17^Department of Hematology and Cell Therapy, Aichi Cancer Center, Nagoya, Japan; ^18^Department of Hematology, Shaare Zedek Medical Center, Faculty of Medicine, Hebrew University of Jerusalem, Israel; ^19^Australian Center for Blood Diseases, The Alfred Hospital and Monash University, Melbourne, Australia; ^20^AbbVie Inc., North Chicago, IL, USA; ^21^Genentech Inc., South San Francisco, CA, USA; ^22^Department of Oncology, Hematology and Bone Marrow Transplantation With Section Pneumology, Hubertus Wald University Cancer Center, University Medical Center Hamburg-Eppendorf, Hamburg, Germany

**Supplemental tables and figures as referenced in the main text are provided below:**

**Supplemental Table 1. Baseline demographics and clinical characteristics of patients in both trials**

| **Characteristics** | **Viale-A*** | | | **Viale-C**† | | |  |
| --- | --- | --- | --- | --- | --- | --- | --- |
|  | **VEN+AZA** | **PBO+AZA** | | **VEN+LDAC** | **PBO+LDAC N=68** | |  |
|  | **N=286** | **N=145** | | **N=143** |  |  |  |
| **Age, median (range), years** | 76 (49–91) | | 76 (60–90) | 76 (36–93) | | 76 (41–88) |  |
| ≥75 years, n (%) | 174 (61) | | 87 (60) | 82 (57) | | 40 (59) |  |
| **Male, n (%)** | 172 (60) | | 87 (60) | 78 (55) | | 39 (57) |  |
| **AML type, n (%)** |  | |  |  | |  |  |
| De novo | 214 (75) | | 110 (76) | 85 (59) | | 45 (66) |  |
| Secondary | 72 (25) | | 35 (24) | 58 (41) | | 23 (34) |  |
| **Secondary AML, n/N (%)** |  | |  |  | |  |  |
| Therapy-related AML | 26/72 (36) | | 9/35 (26) | 6/58 (10) | | 4/23 (17) |  |
| History of MDS or CMML/prior hematologic disorder | 46/72 (64) | | 26/35 (74) | 52/58 (90) | | 19/23 (83) |  |
| **ECOG performance status, n (%)** |  |  |  |  |  |  |  |
| 0–1 | 157 (55) | | 81 (56) | 74 (52) | | 34 (50) |  |
| 2–3 | 129 (45) | | 64 (44) | 69 (48) | | 34 (50) |  |
| **Prior HMA treatment, n (%)** | – | | – | 28 (20) | | 14 (21) |  |
| **Cytogenetic risk, n (%)** |  | |  |  | |  |  |
| Favorable | – | | – | 1 (1) | | 3 (4) |  |
| Intermediate | 182 (64) | | 89 (61) | 90 (63) | | 43 (63) |  |
| Poor | 104 (36) | | 56 (39) | 47 (33) | | 20 (29) |  |
| *DiNardo CD, et al. N Engl J Med. 2020;383(7):617-29. †Wei AH, et al. Blood. 2020 Jun 11;135(24):2137-2145. AML, acute myeloid leukemia; AZA, azacitidine; CMML, chronic myelomonocytic leukemia; ECOG, Eastern Cooperative Oncology Group; HMA, hypomethylating agent; LDAC, low-dose cytarabine; MDS, myelodysplastic syndromes; PBO, placebo; RBC, red blood cell; VEN, venetoclax. | | | | | | |  |
|  |  |  |  |  |  |  |  |
|  |  |  |  |  |  |  |  |
|  |  |  |  |  |  |  |  |
|  |  |  |  |  |  |  |  |

**Kaplan-Meier Curves from subgroup analyses**

**Supplemental Figure 1. Time to deterioration in health status VAS (A) and EORTC GHS/QoL subgroup analyses in patients with CR + CRi in Viale-A**

**A.**

**
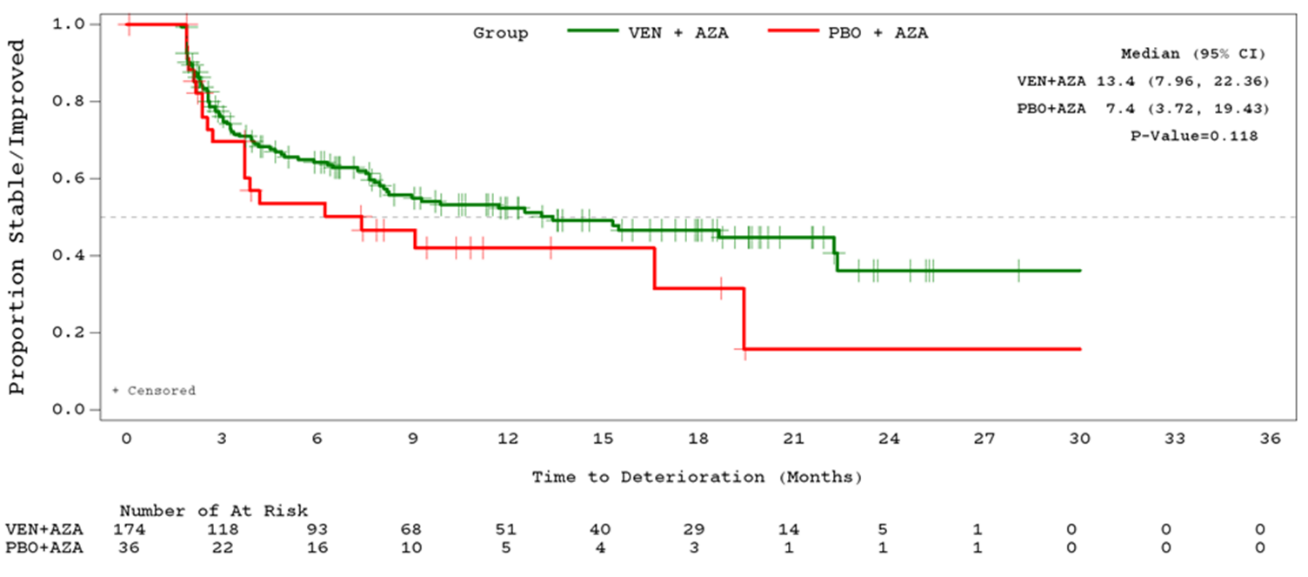
**


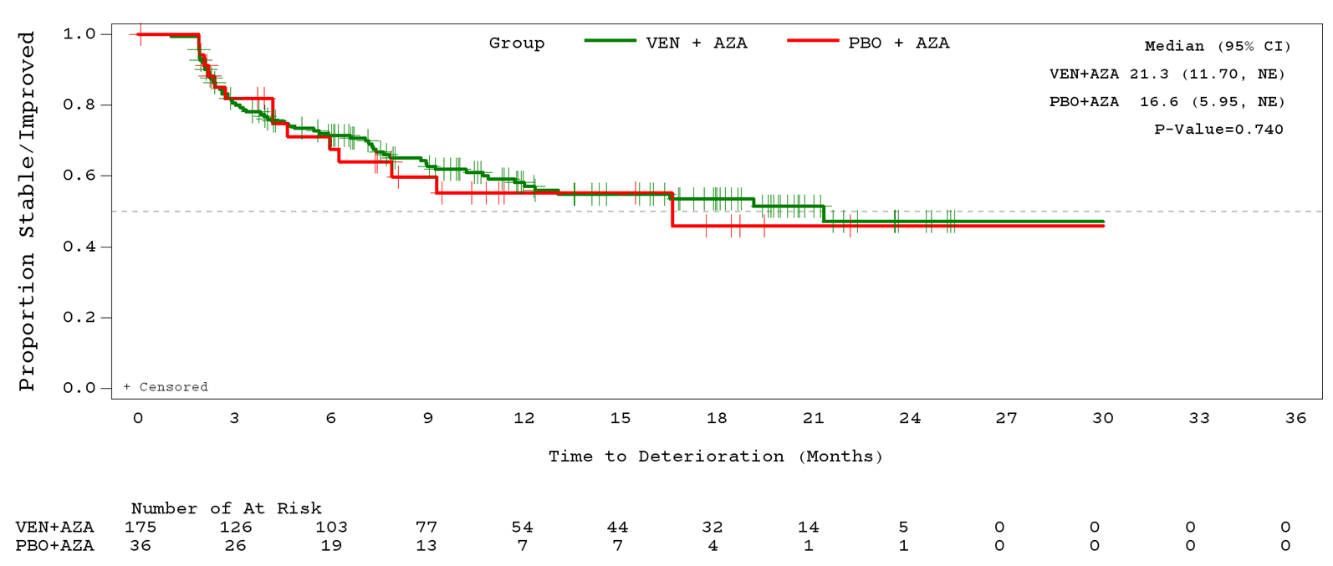
**B.**

AZA, azacytidine; CI, confidence intervals; CR, complete remission; CRi, complete remission with incomplete blood count recovery; EORTC QLQ-C30, European Organisation for Research and Treatment of Cancer quality of life questionnaire; GHS, global health status; PBO, placebo; QoL, quality of life; VAS, visual analog scale; VEN, venetoclax.

**Supplemental Figure 2. Time to deterioration in EORTC-GHS/QoL among patients age <75 years in Viale-A (A) and Viale-C (B)**

**A.**

**
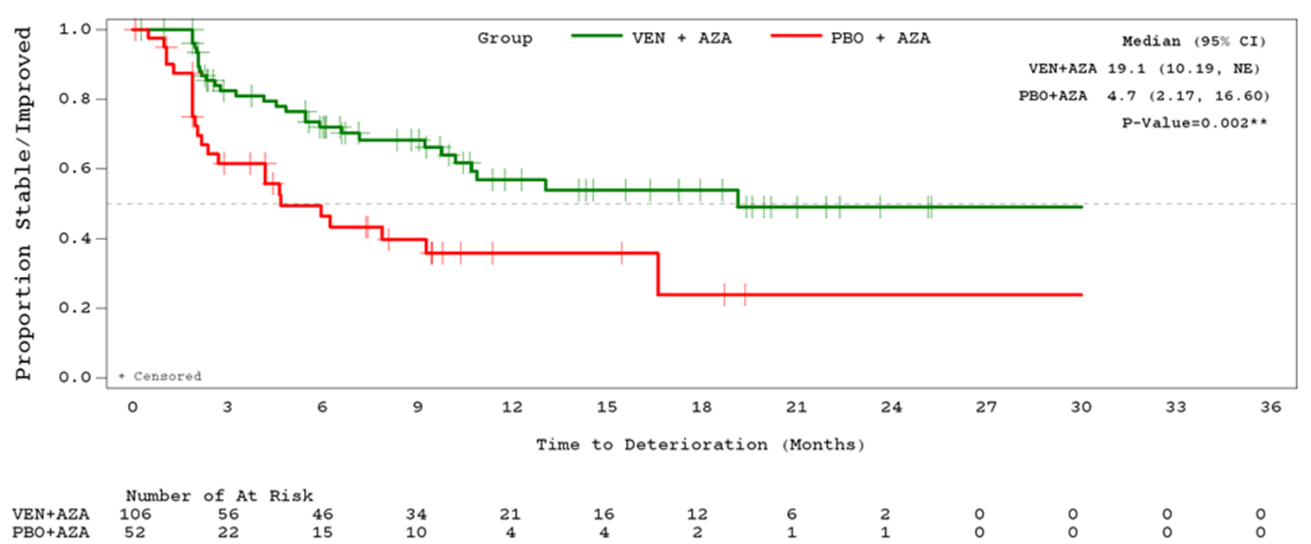
**

**B.**


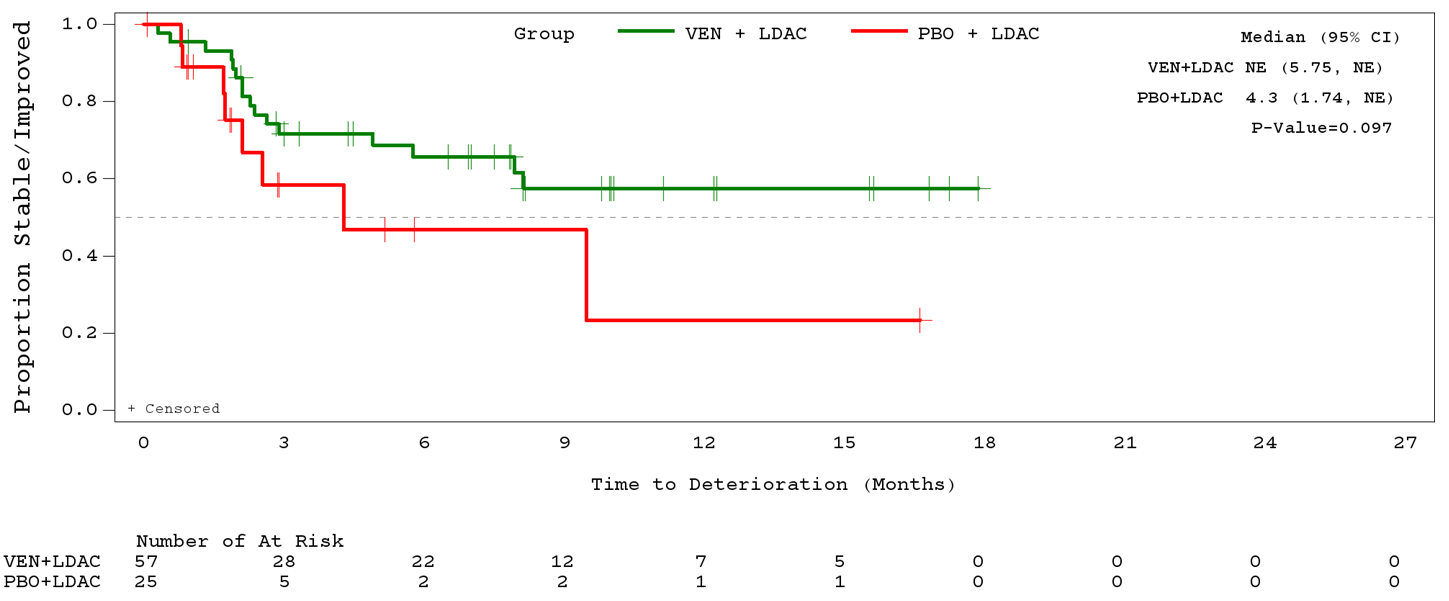


AZA, azacytidine; CI, confidence intervals; EORTC QLQ-C30, European Organisation for Research and Treatment of Cancer quality of life questionnaire; GHS, global health status; LDAC, low-dose cytarabine; NE, not estimable; PBO, placebo; QoL, quality of life; VEN, venetoclax.

**Supplemental Figure 3. Viale-A time to deterioration in EORTC-GHS/QoL among patients age ≥75 years in Viale-A (A) and Viale-C (B)**

**A.**

**
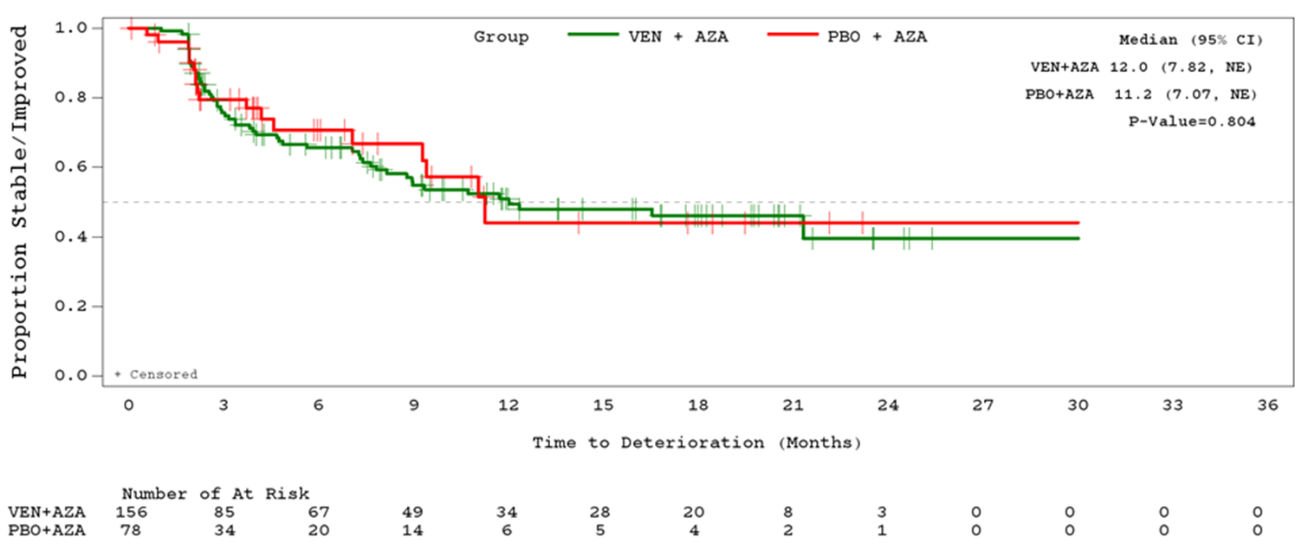
**

**B.**


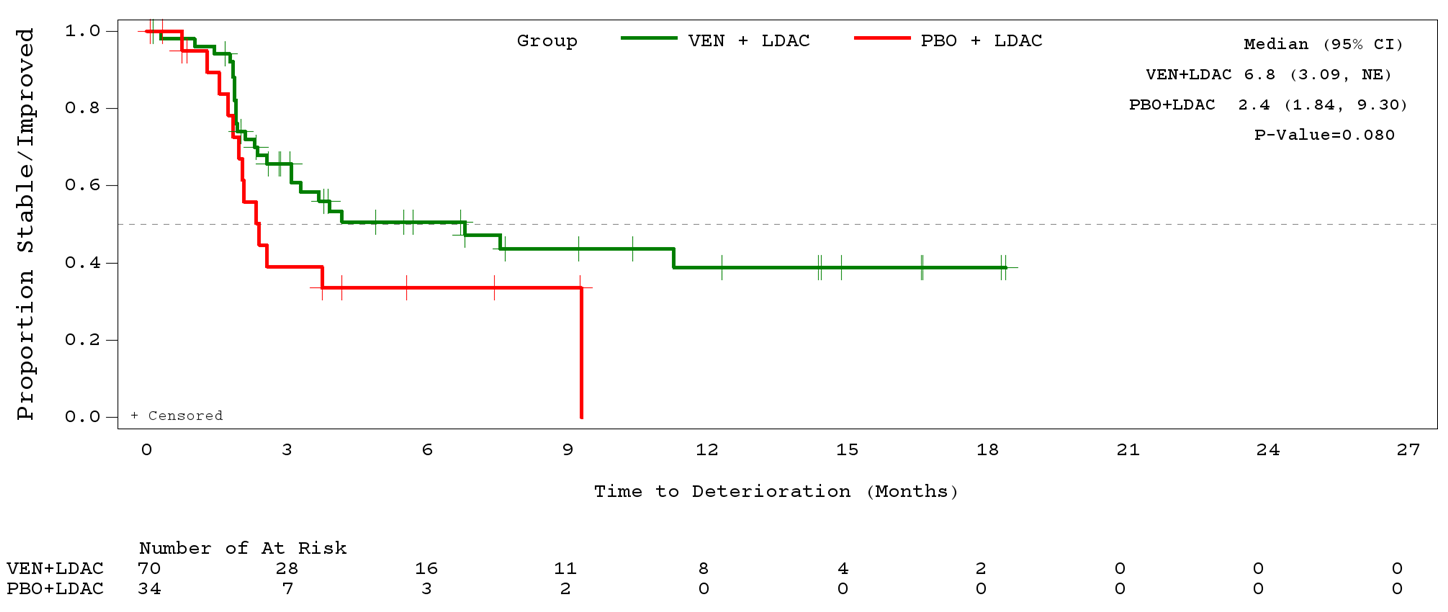


AZA, azacytidine; CI, confidence intervals; EORTC QLQ-C30, European Organisation for Research and Treatment of Cancer quality of life questionnaire; GHS, global health status; LDAC, low-dose cytarabine; NE, not estimable; PBO, placebo; QoL, quality of life; VEN, venetoclax.

**Supplemental Figure 4. Time to deterioration in EORTC-GHS/QoL among patients with post-baseline TI RBC in Viale-A (A) and Viale-C (B)**

**A**

**
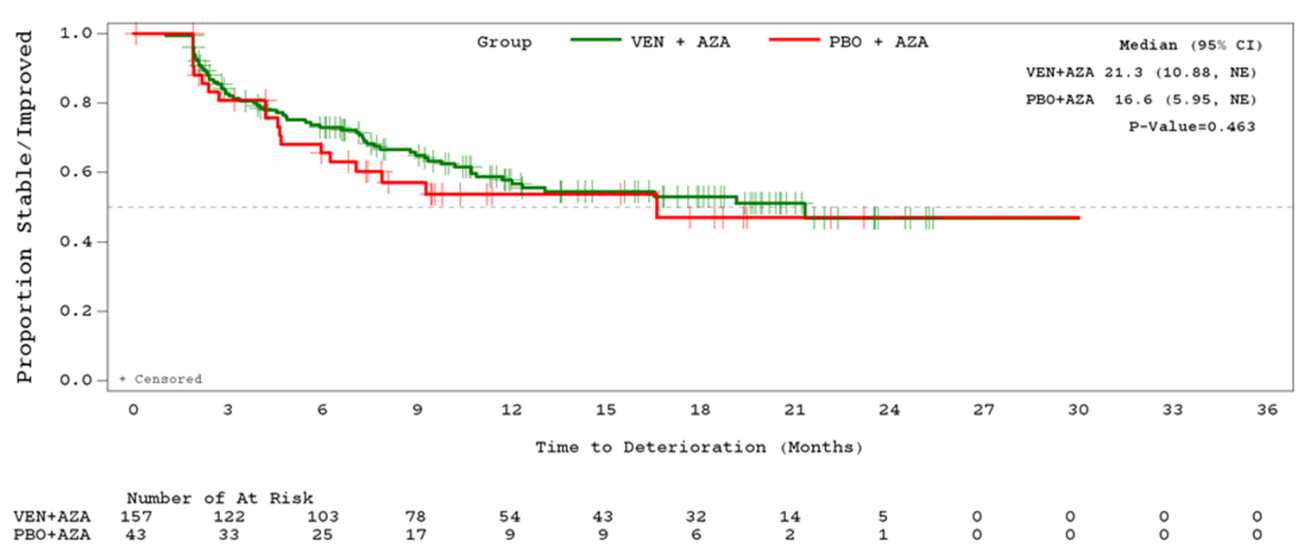
**

**B**

**
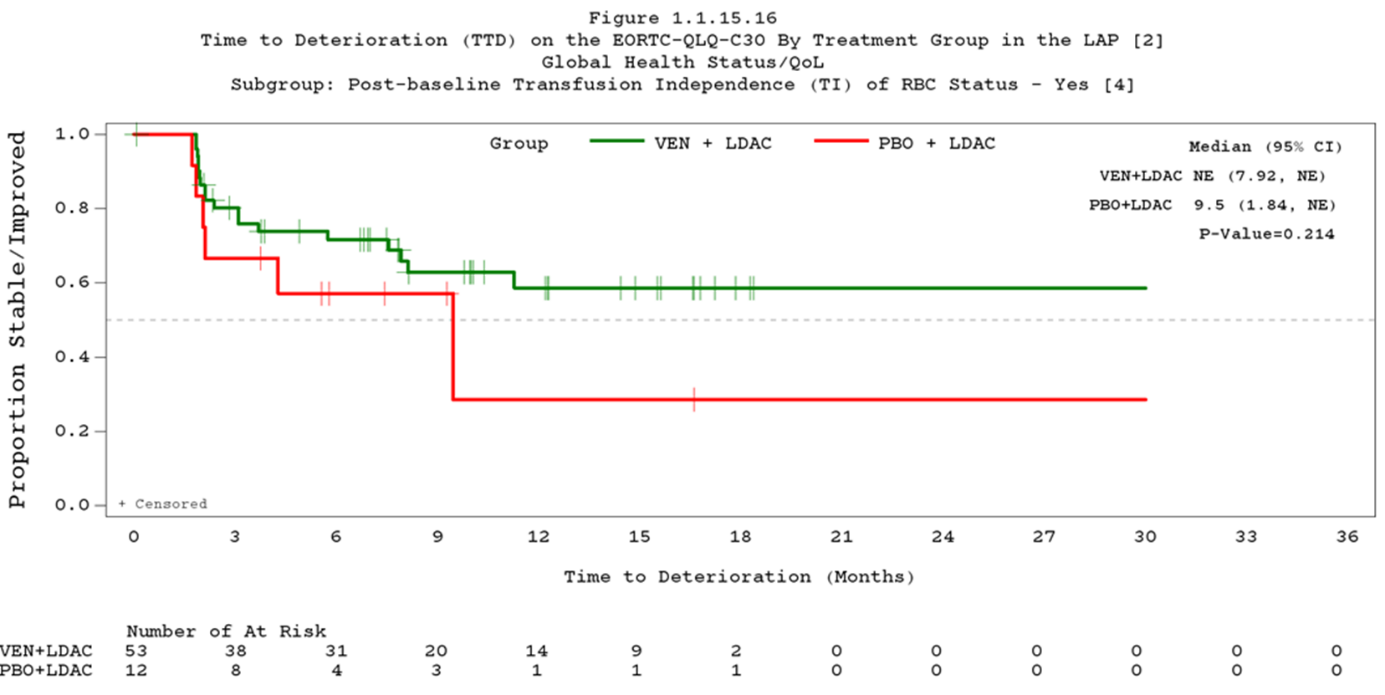
**

AZA, azacytidine; CI, confidence intervals; EORTC QLQ-C30, European Organisation for Research and Treatment of Cancer quality of life questionnaire; GHS, global health status; LDAC, low-dose cytarabine; NE, not estimable; PBO, placebo; QoL, quality of life; RBC, red blood cells; TI, transfusion independence; VEN, venetoclax.

**Supplemental Figure 5. Time to deterioration in EORTC-GHS/QoL among patients with post-baseline TI platelets in Viale-A (A) and viale-C (B)**

**A**

**
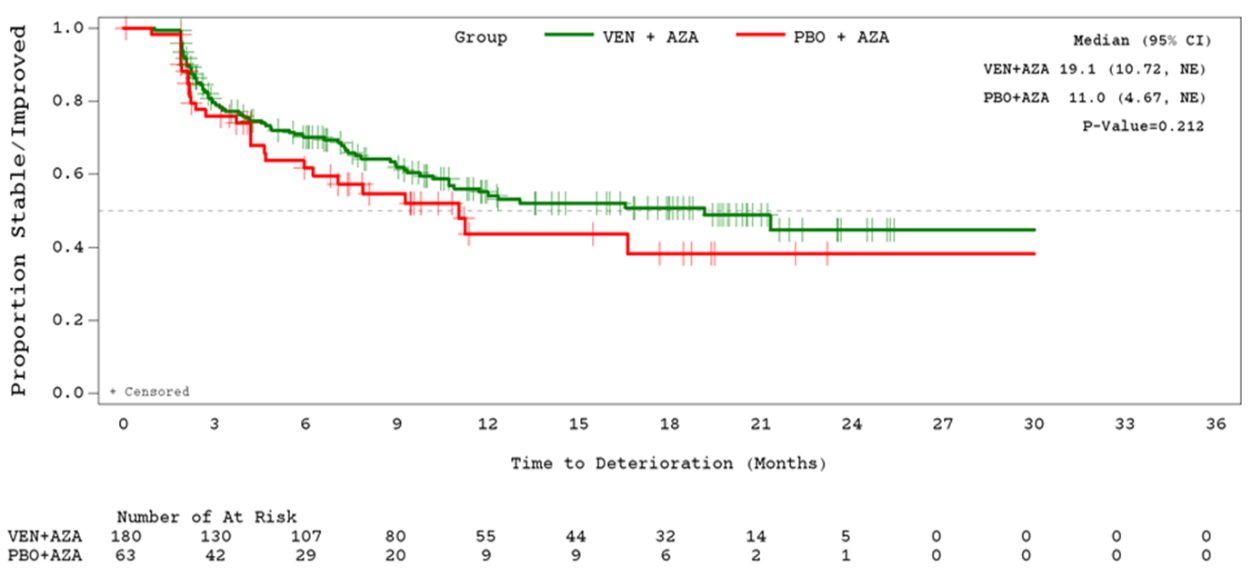
**

**B**

**
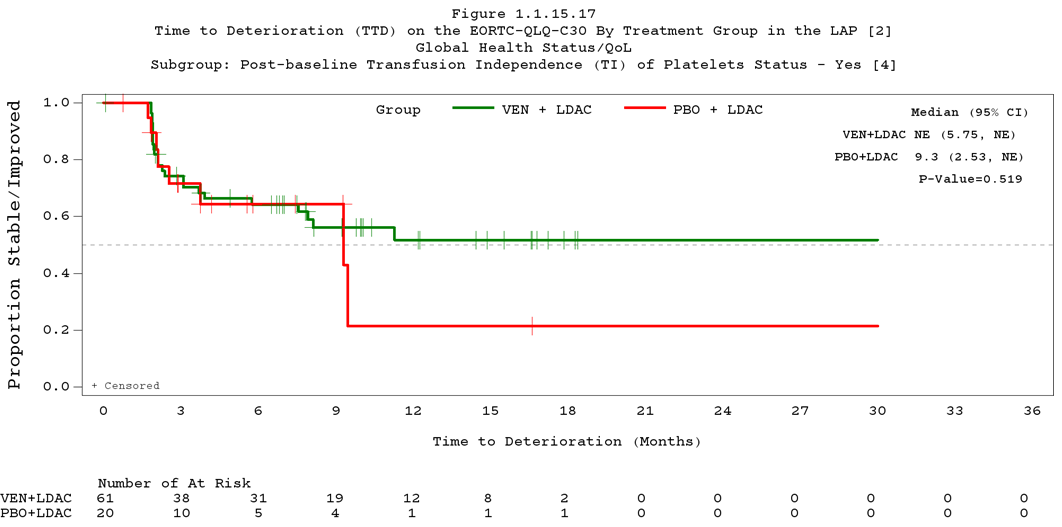
**

AZA, azacytidine; CI, confidence intervals; EORTC QLQ-C30, European Organisation for Research and Treatment of Cancer quality of life questionnaire; GHS, global health status; LDAC, low-dose cytarabine; NE, not estimable; PBO, placebo; QoL, quality of life; TI, transfusion independence; VEN, venetoclax.

**Supplemental Figure 6. Time to deterioration in EORTC-GHS/QoL among patients with baseline ECOG scores >2 in Viale-A (A) and Viale-C (B)**

**A.**


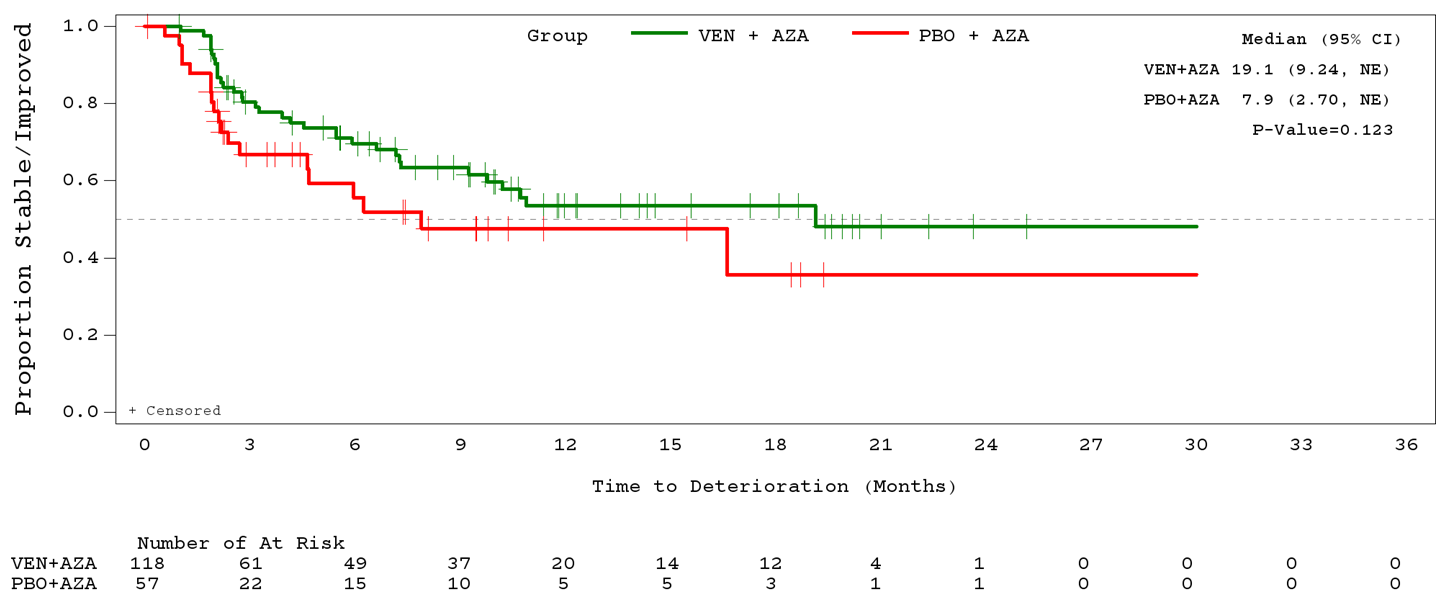


**B.**


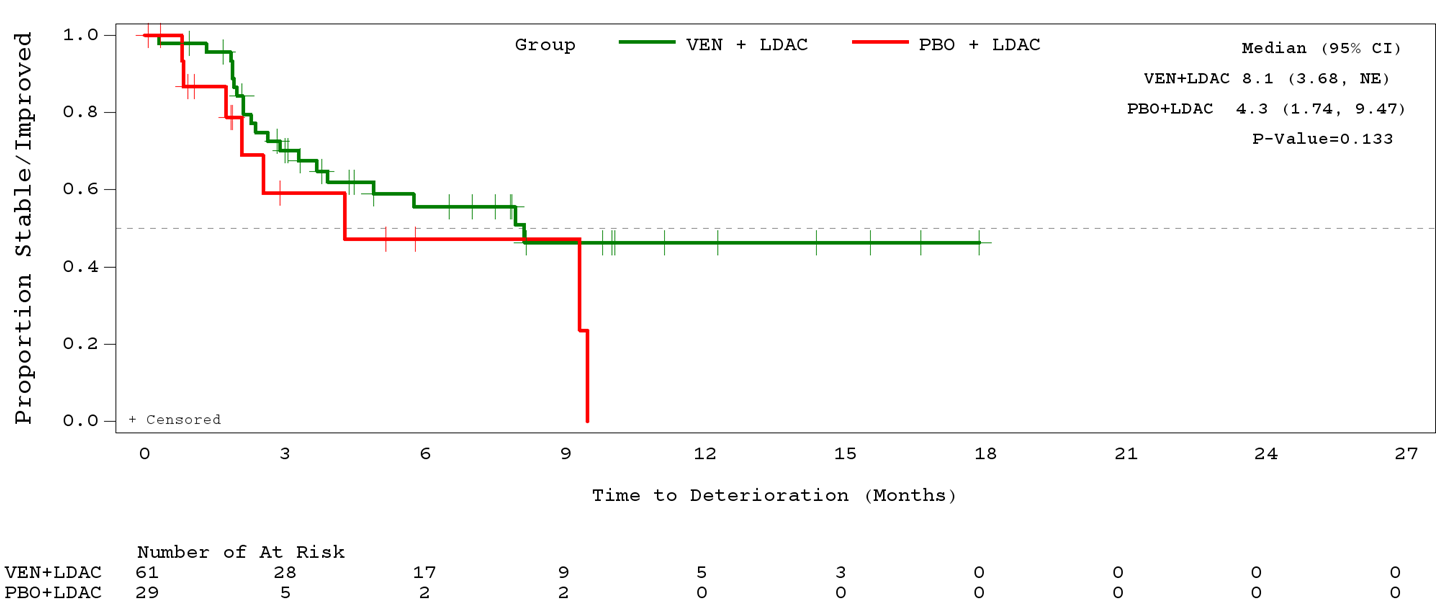


AZA, azacytidine; CI, confidence intervals; ECOG, Eastern Cooperative Oncology Group; EORTC QLQ-C30, European Organisation for Research and Treatment of Cancer quality of life questionnaire; GHS, global health status; LDAC, low-dose cytarabine; NE, not estimable; PBO, placebo; QoL, quality of life; VEN, venetoclax.

**Kaplan-Meier curves sensitivity analyses**

**Supplemental Figure 7. Viale-A sensitivity analysis**

1. **Progression, death or EORTC QLQ-C30 GHS/QoL – Time to deterioration of composite events**

**
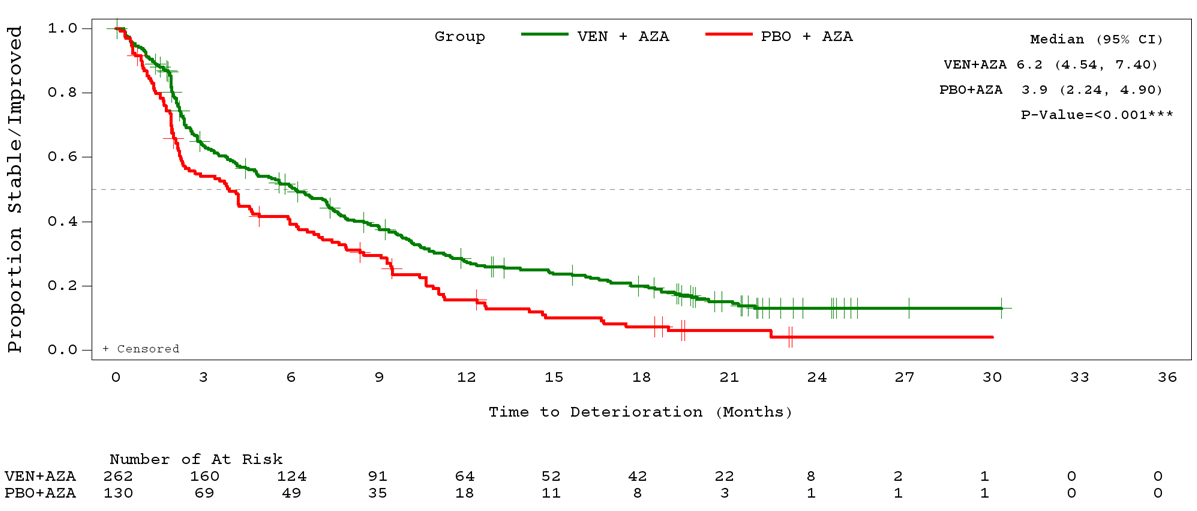
**

Composite events: Progressive disease,* death or deterioration in PRO MCT threshold EORTC QLQ-C30 GHS/QoL MCT =10, whichever occurred first

**
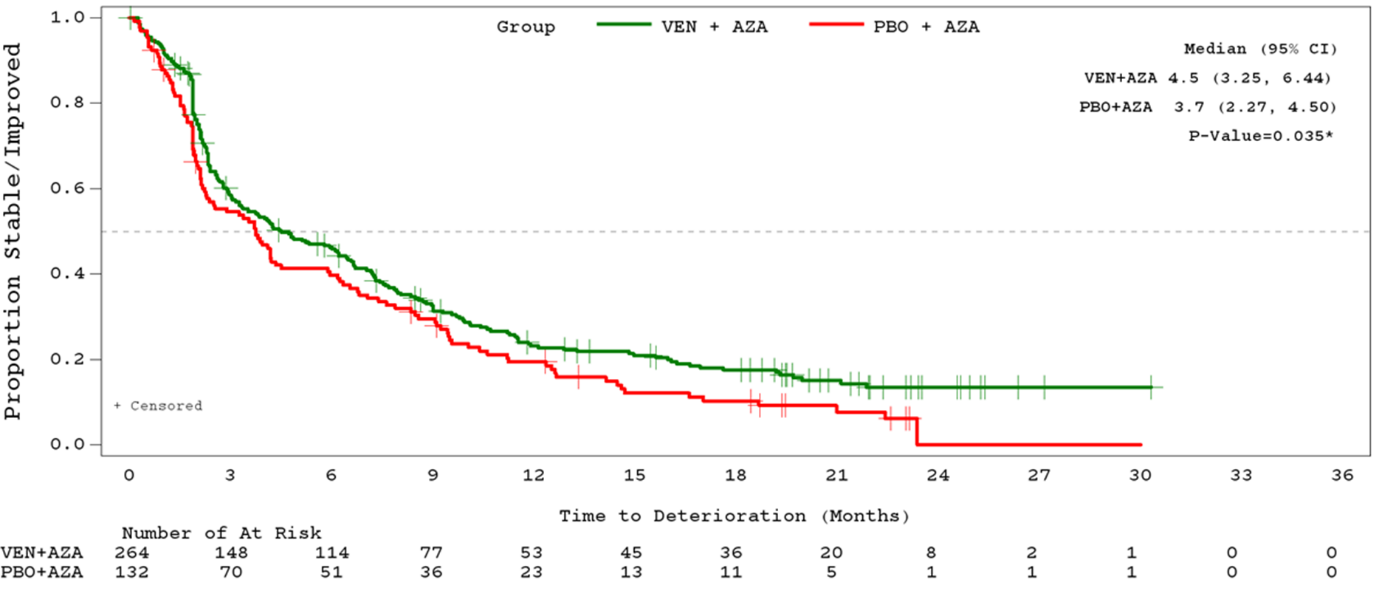
B. Progression, death or PROMIS Fatigue – Time to deterioration of composite events**

Composite events: progressive disease,* death or deterioration in PRO MCT threshold PROMIS Fatigue =5, whichever occurred first

**C. Progression, death or EORTC QLQ-C30 PF– Time to deterioration of composite events**


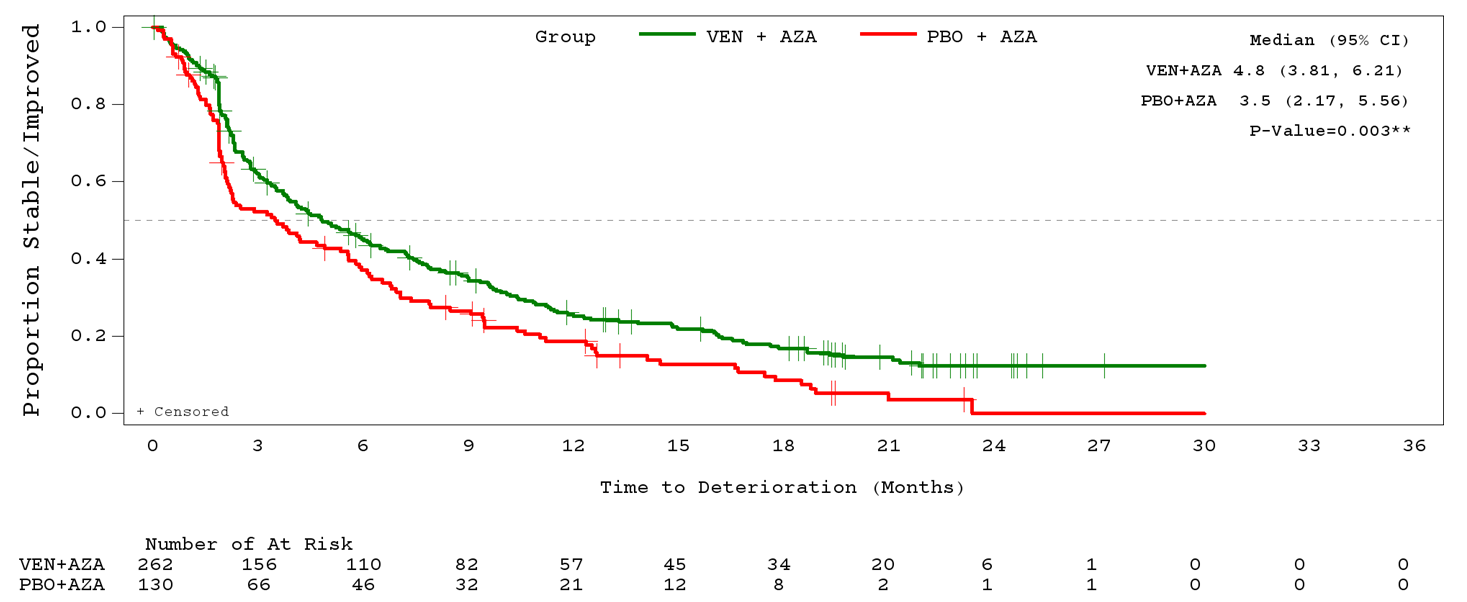


Composite events: Progressive disease,* death or deterioration in PRO MCT threshold EORTC QLQ-C30 PF MCT =10, whichever occurred first

**
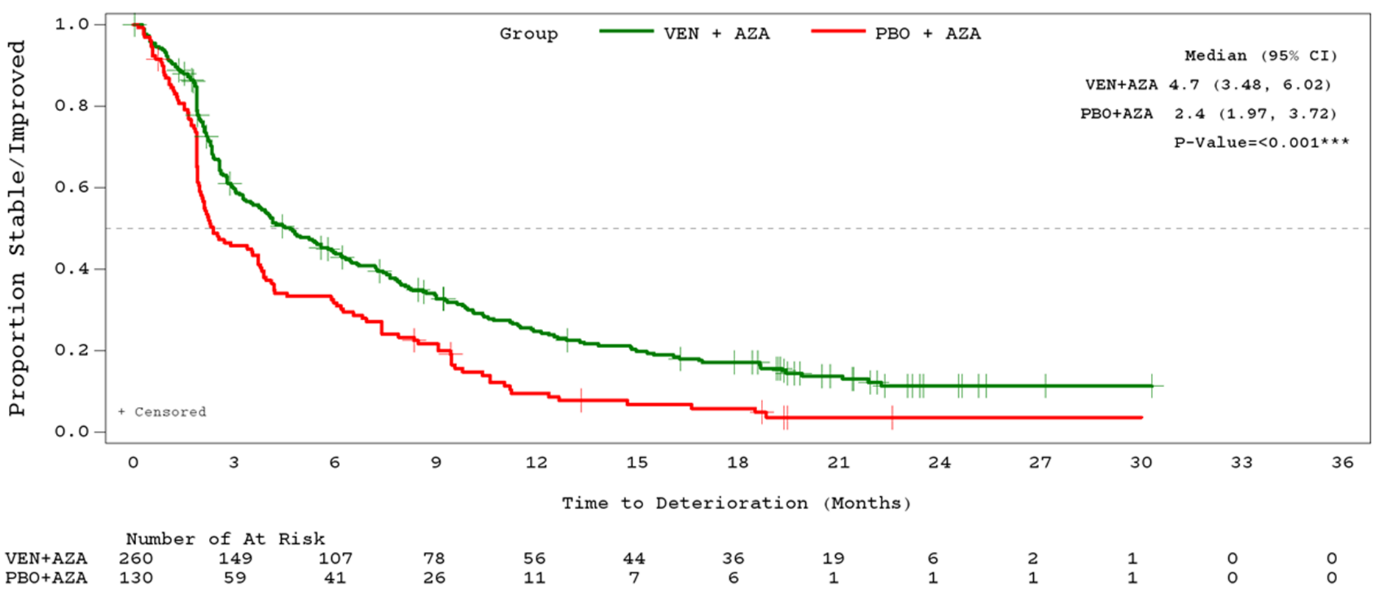

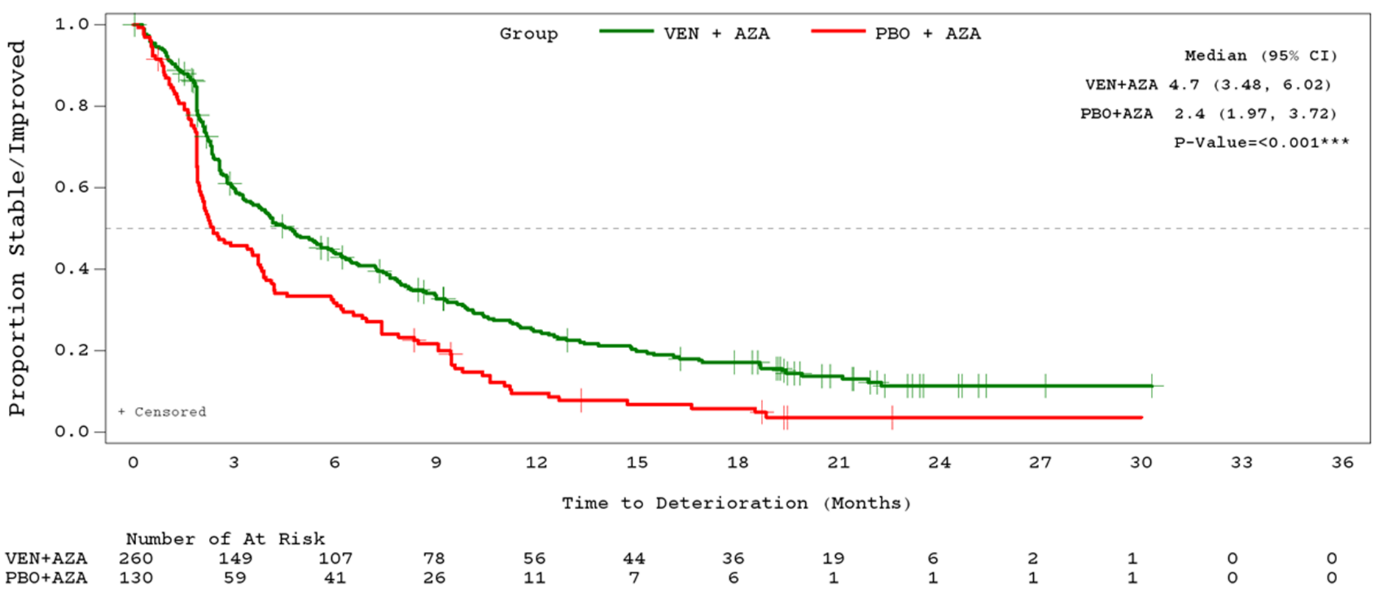
D. Progression, death or EQ-5D-5L health status VAS - Time to deterioration of composite events**

Composite events: progressive disease,* death or deterioration in PRO MCT threshold EQ-5D-5L health status MCT =7, whichever occurred first

*Progressive disease or death will be derived from event-free survival (EFS). EFS will be defined as the number of days from randomization to the date of progressive disease, relapse from CR or CRi, investigator assessed treatment failure or death from any cause. Treatment failure will be defined as failure to achieve CR, CRi, PR or morphologic leukemia free state (MLFS).
AZA, azacytidine; CI, confidence intervals; CR, complete remission; CRi, complete remission with incomplete blood count recovery**;** EORTC QLQ-C30, European Organisation for Research and Treatment of Cancer quality of life questionnaire; EQ-5D-5L, EuroQoL 5-Dimension 5-Level; GHS, global health status; PBO, placebo; PF, physical functioning; PROMIS, Patient-Reported Outcomes Measurement Information System; QoL, quality of life; VAS, visual analog scale; VEN, venetoclax.

**Supplemental Figure 8. Viale-C sensitivity analysis**

**A. Progression, death or EORTC QLQ-C30 GHS/QoL - Time to deterioration of composite events**

**
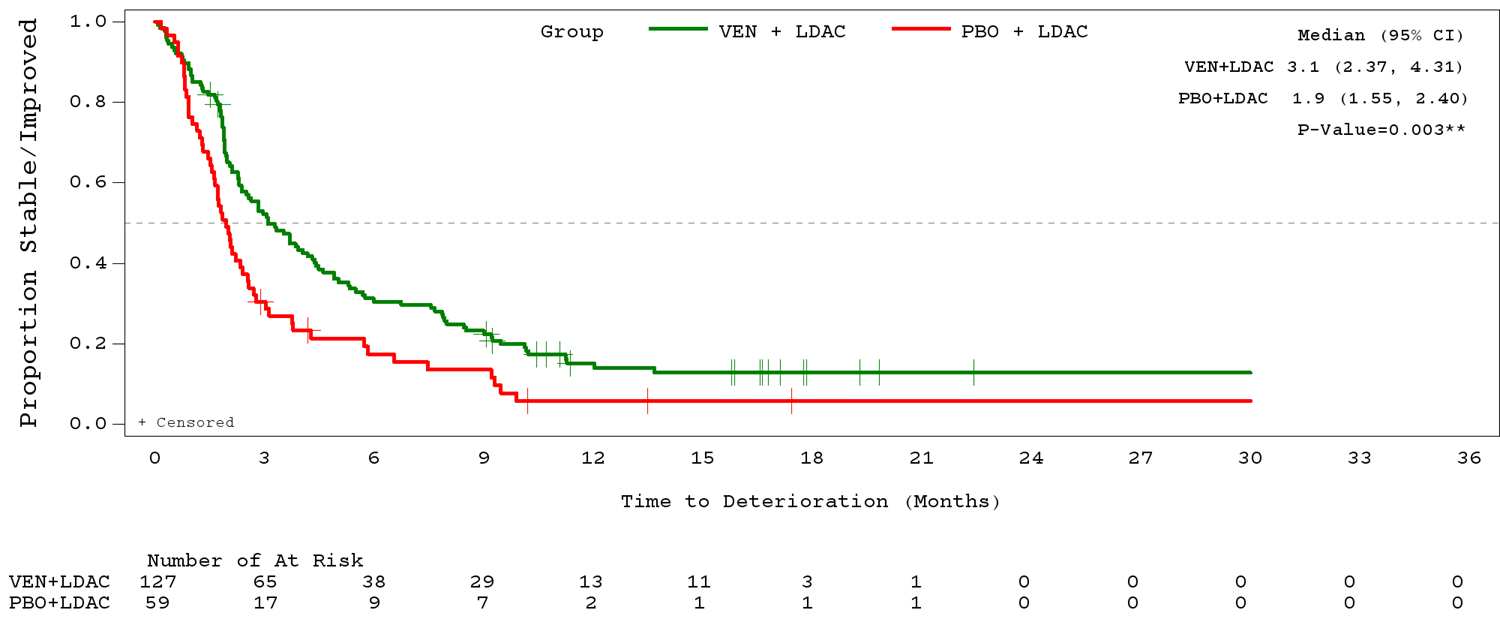
**

Composite events: progressive disease,* death or deterioration in PRO MCT threshold EORTC QLQ-C30 GHS/QoL MCT =10, whichever occurred first

**
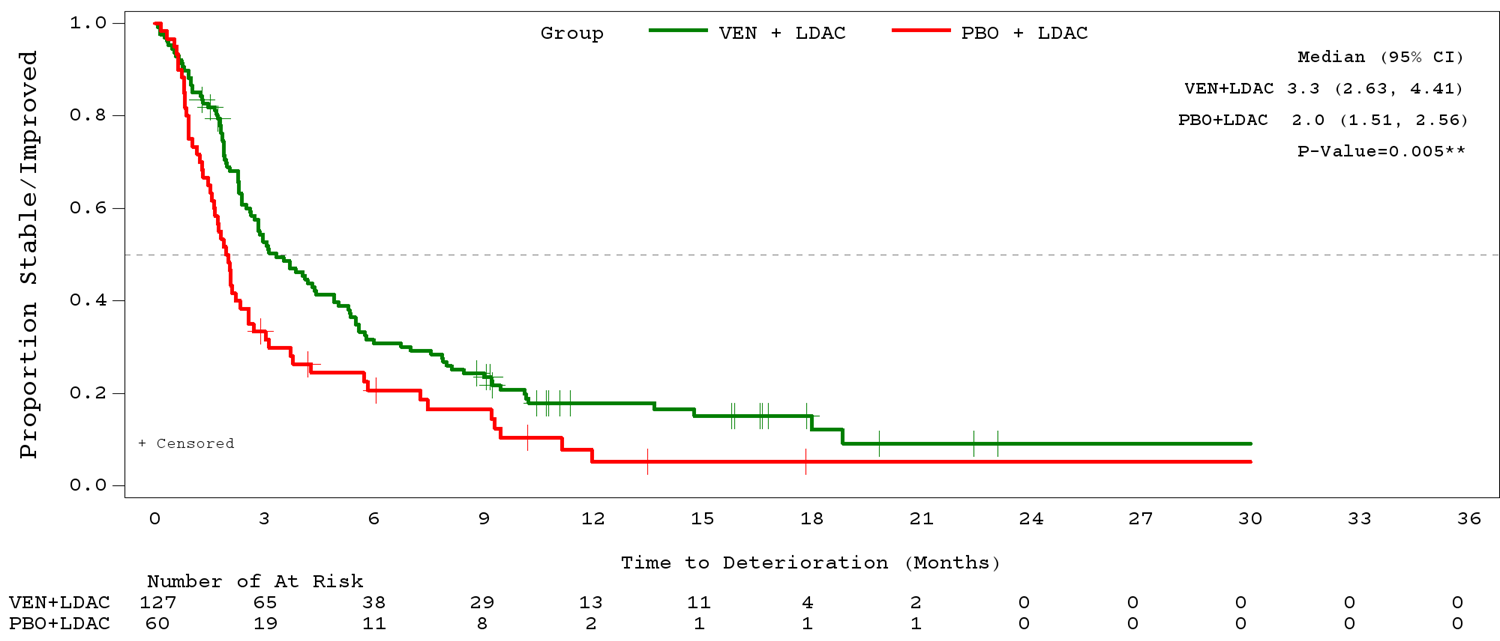
B. Progression, death or PROMIS Fatigue - Time to deterioration of composite events**

Composite events: progressive disease,* death or deterioration in PRO MCT threshold PROMIS Fatigue =5, whichever occurred first

**C. Progression, death or EORTC QLQ-C30 PF– Time to deterioration of composite events**


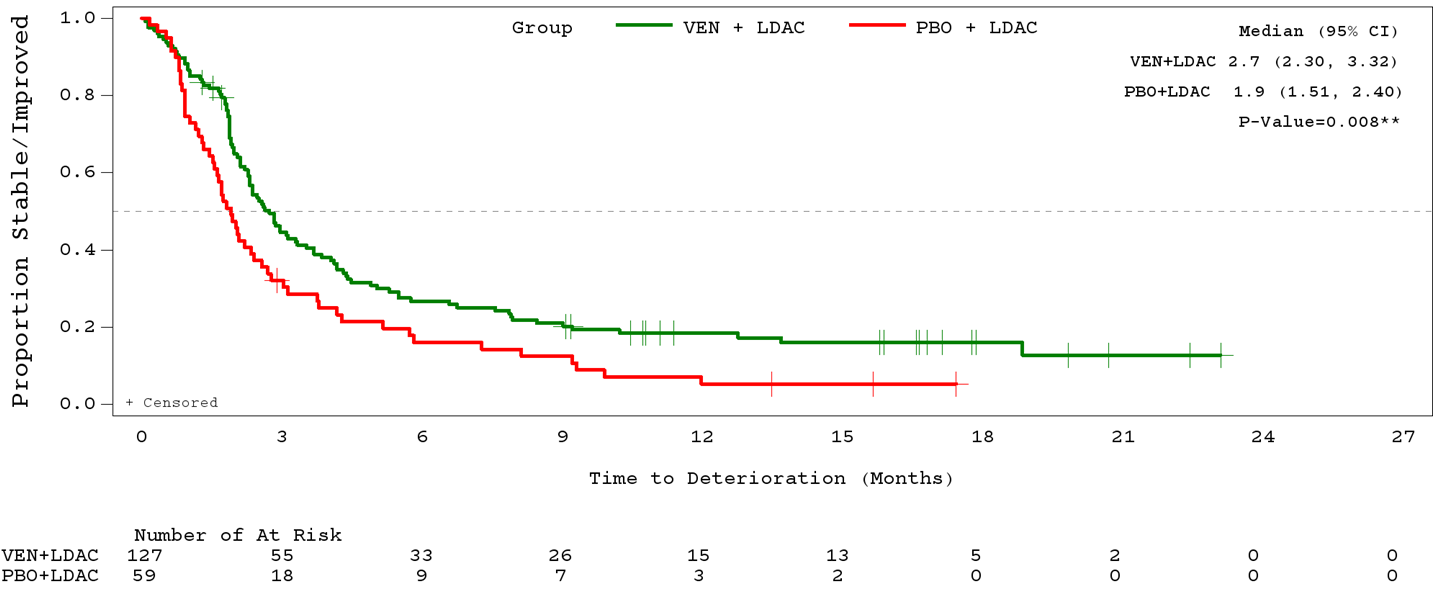


Composite events: Progressive disease,* death or deterioration in PRO MCT threshold EORTC QLQ-C30 PF MCT =10, whichever occurred first

**D. Progression, death or EQ-5D-5L health status VAS - Time to deterioration of composite events**

**
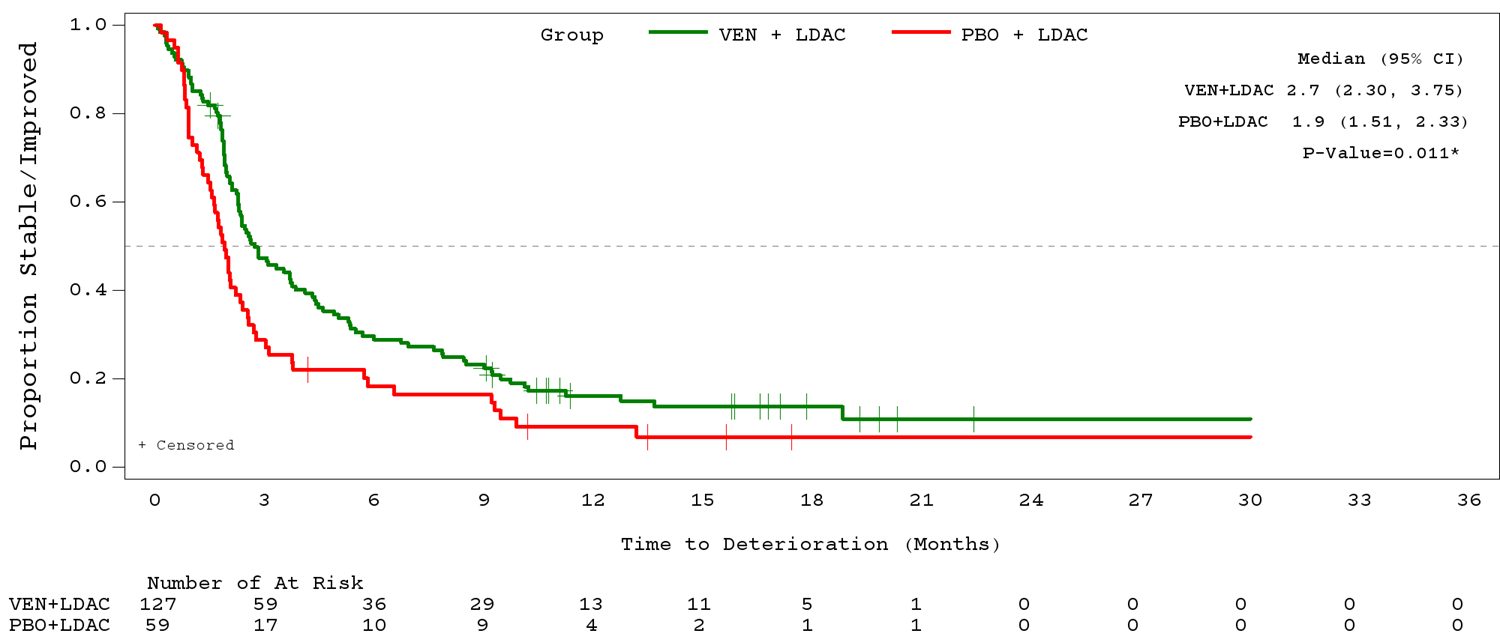
**

Composite events: Progressive disease,* death or deterioration in PRO MCT threshold EQ-5D-5L health status MCT =7, whichever occurred first

*Progressive disease or death will be derived from event-free survival (EFS). EFS will be defined as the number of days from randomization to the date of progressive disease, relapse from CR or CRi, investigator assessed treatment failure or death from any cause. Treatment failure will be defined as failure to achieve CR, CRi, PR or morphologic leukemia free state (MLFS).
CI, confidence intervals; CR, complete remission; CRi, complete remission with incomplete blood count recovery**;** EORTC QLQ-C30, European Organisation for Research and Treatment of Cancer quality of life questionnaire; EQ-5D-5L, EuroQoL 5-Dimension 5-Level; GHS, global health status; LDAC, low-dose cytarabine; PBO, placebo; PROMIS, Patient-Reported Outcomes Measurement Information System; QoL, quality of life; VAS, visual analog scale; VEN, venetoclax.

**Additional information on patient-reported outcome measures utilized**

European Organisation for Research and Treatment of Cancer quality of life questionnaire (EORTC QLQ-C30) instrument (including the global health status and physical functioning domains)

- *General information:* <https://qol.eortc.org/questionnaire/eortc-qlq-c30/>
- *Scoring manual:* <https://www.eortc.org/app/uploads/sites/2/2018/02/SCmanual.pdf>
- *Sample assessment form:* <https://www.eortc.org/app/uploads/sites/2/2018/08/Specimen-QLQ-C30-English.pdf>

Patient-Reported Outcomes Measurement Information System Short Form Fatigue 7a instrument

- *General information, scoring manual, and sample assessment form*: <https://www.healthmeasures.net/index.php?option=com_instruments&view=measure&id=160&Itemid=992>

EuroQoL 5-dimension 5-level instrument (assessed on visual analog scale)

- *General information:* <https://euroqol.org/eq-5d-instruments/eq-5d-5l-about/>
- *Scoring manual:* <https://euroqol.org/publications/user-guides/>
- *Sample assessment form:* <https://euroqol.org/eq-5d-instruments/sample-demo/>
